# Supplementary material for: Improving quality of surgical and anaesthesia care at hospital level in sub-Saharan Africa: a systematic review protocol of health system strengthening interventions
Source: BMJ Open. 2020 May 30;10(5):e036615. doi: 10.1136/bmjopen-2019-036615 (PMC7264698; doi:10.1136/bmjopen-2019-036615)
Supplement: Supplementary data [file bmjopen-2019-036615supp002.pdf]

Appendix1: Search strategies for MEDLINE

1) Surgical or anaesthesia ;2) quality improvement hospital level interventions;3) Sub-Saharan African countries (SSACs)

Search String 1	Search String 2	Search String 3
surg* OR surgic* OR an?esthes* OR operat* OR pre?operat* OR post?operat* OR obstetr* OR laparotomy OR ambulatory surgical procedures/ or exp laparotomy/ or exp minor surgical procedures/ or obstetric surgical procedures/ or exp perioperative care/ or perioperative exp period/ OR exp General Surgery/ OR exp Anesthetics, General/ or exp Anesthetics, Inhalation/ or exp Anesthetics, Combined/ or exp Anesthetics/ or exp	Hospital* OR in?patient* OR ward* OR (intensive adj1 care*).mp OR exp Hospitals/ OR exp Intensive Care Units, Pediatric/ or exp Hospital Units/ or exp Mobile Health Units/ or exp Coronary Care Units/ or exp Intensive Care Units/ or exp Colony-Forming Units Assay/ or exp Burn Units/ or exp Intensive Care Units, Neonatal/ OR exp inpatient/ OR (Operat* adj2 (room* or theatre)).mp<br>AND (any of the following)<br>intersectoral collaboration/ or efficiency, organizational/ or "facilities and services utilization"/ or hospital information systems/ or hospital-patient relations/ or database management systems/ or ambulatory care information systems/ or medical order entry systems/ or operating room information systems/ or point-of-care systems/ or "personnel staffing and scheduling information systems"/ or medication systems, hospital/ or patient identification systems/ or health smart cards/ or radio frequency identification device/ or employee discipline/ or employee incentive plans/ or employee performance appraisal/ or staff development/ or time management/<br>advance directive adherence/ or exp clinical competence/ or exp guideline adherence/ or exp patient reported outcome measures/ or exp alert fatigue, health personnel/ or exp benchmarking/ or exp clinical audit/ or exp credentialing/ or exp "facility regulation and control"/ or exp guidelines as topic/ or exp Quality Control/ or exp "Quality of Life"/ or exp Quality-Adjusted Life Years/<br>OR "Reminder Systems"/ or "Total Quality Management"/ or exp "Patient Education"/ or "Health Education"/ or exp "Consumer Health Information"/ or "Critical Pathways"/ or "Education, Medical, Continuing"/ or exp "Inservice Training"/ or "Guideline Adherence"/ or "Clinical Competence"/ or "Peer Review"/ or exp "Medical Audit"/ OR OR patient education.mp OR critical pathway*.mp OR clinical pathway*.mp OR care pathway*.mp OR (continuing adj2 education).mp OR (inservice or in service).mp OR (staff adj3 train*).mp OR audit* or feedback* OR exp Medical Record Linkage/ OR exp Patient Safety/ or exp Safety Management/ OR "task performance and analysis"/ or "time and motion studies"/ or work simplification/ OR	sub?Saharan Africa*.mp OR Angola or Benin or Bolivia or "Burkina Faso" or Burundi or "Cabo Verde" or Cameroon or "Central African Republic" or Chad or Comoros or Congo or "Democratic Republic of the Congo" or DRC or "Republic of the Congo" or "Cote d'Ivoire" or "Ivory Coast" or "Equatorial Guinea" or Eritrea or Eswatini or Ethiopia or Gabon or Gambia or Ghana or Guinea or "Guinea?Bissau" or Kenya or Lesotho or Liberia or Madagascar or Malawi or Mali or Mauritania or Mauritius or Mozambique or Namibia or Niger or

Anesthetics, Local/ or exp Anesthetics, Intravenous/ or exp Anesthetics, Dissociative/ OR exp Obstetrics/	"patient risk management".mp OR “clinical documentation” OR ”nursing documentation” OR (clinical adj2 record*) OR (patient* adj2 record*) OR (hospital adj2 documentation) OR (nursing process) OR (Patient adj2 care plan*) OR (nurs* adj2 documentation adj2 framework*) OR<br>OR (audit adj3 feedback).mp OR (safety checklist* or safety check list*).mp, (clinical adj2 record*).mp, (patient* adj2 record*).mp, (clinical adj2 documentation).mp, (data adj2 qualit*).mp, (hospital adj2 documentation).m<br>AND<br>exp Quality Improvement/ OR "Outcome and Process Assessment"/ or "Outcome Assessment"/ or "Process Assessment"/ or "Quality of Health Care"/ or "Quality Assurance"/ or "Quality Improvement"/ or "Quality Indicators"/ or "Management Quality Circles" OR (outcome* adj3 assessment*).mp OR (process* adj3 assessment*).mp OR quality assurance.mp OR quality improvement.mp OR (improvement adj intervention*).mp OR (improvement adj program*).mp OR (improvement adj initiative*).mp OR (process* adj improvement).mp OR quality indicator*.mp OR quality circle*.mp OR (total quality management or six sigma*).mp OR (program* adj3 effectiveness).mp OR (program* adj3 evaluation*).mp OR "Program Evaluation"/ or quality* or safet*	Nigeria or Rwanda or "Sao Tome and Principe" or Senegal or "Sierra Leone" or Seychelles or Somalia or "South Africa" or "South Sudan" or Sudan or Tanzania or Togo or Uganda or Zambia or Zimbabwe

*Include USA spelling, plurals, and synonym

Appendix2: Database literature search log.

Date	Database Name	Search Terms	Search Limits	Results	Comments

Apendix3: Systematic Review Abstract and Full Text Screening Criteria

Topic	Criteria	Yes	Unclear	No
Type of article	Is this a Peer-reviewed research articles			
Health Intervention care setting (HICS)	Is this intervention in hospital-based setting			
Type of condition	1.Does this study relate to any surgical and anaesthesia care presentation			
	2.Does this study relate to trauma/injury care, cosmetic surgery or sport medicine?			
Outcome	Does this study report Outcomes-Clinicla, Process, Implementation			
Subject of study	Does this study about Medical production, vaccines and technologies			

Instruction

- Include Abstract and Find Full Text if Type of article (Yes) AND HICS (Yes) AND Type of conditions 1 (Yes) AND Type of conditions 2 (No/Unclear) AND Outcome (Yes) and Subject Study (No)
- Exclude Abstract and Full text if Type of article (Unclear/No) OR HICS (Unclear/No) OR Type of conditions 1 (Unclear/No) OR Type of conditions 2 (Yes) OR Outcome (No) OR Subject Study (Yes)

Appendix 4: Systematic Review Data Extraction Form

Variable	Response
**Reference**	[text]
**Year**	[number]
**Language**	[text]
**Location(s)**	[text]
**Type of publication**	[Grey (1) /Postgrad Dissertation (2)/Published- peer reviewed (3)/Non-research report (4)]
**Journal**	[text]
**Journal Field**	[text]
**Name of low and middle income country**	[text]
**Brief description of study**	[text]
**Surgical and anaesthesia care Intervention**	[yes/no]
**Integrated into routine health services**	[yes/no]
**Health care setting**	[Tertiary (1)/ Secondary Hospital (2)/ Level-hospital department (triage, surgical theatre) (3)]
**Study Methodology**	[Qualitative (1)/quantitative (2)]/ mixed (3)
**Study design**	[RCT, Observational, cohort, quazi-experimental, etc)]
**Describes Evaluation of the intervention**	[retrospective/prospective/neither]
**Type of health intervention**	[Service delivery (1)/Health Workforce (2)/Information (3)/ Financing (4)/Leadership/Governance (5)]
**Population size/Sample size**	
**Primary clinical outcome**	[mortality Yes/No]
**Result of Primary clinical Outcome**	[test]
**Secondary Clinical outcome collected and assessed**	[major or minor complications, SSI, etc]
**Process outcomes collected and assessed**	[text]
**Implementation outcome collected and assessed**	[one of 8 as defined by Proctor]
**Other outcomes collected and assessed**	[text]
**Describes design of intervention**	[text] e.g System thinking, TOC, LF
**Describes Implementation of intervention**	[text] e.g Fidelity, Adoption
**Type of impact**	[text]
**Detail on statistical analysis**	[text]
**Potential Biases or concerns**	[text]
**Cost/source of funding for intervention**	
**Other points or key lessons for reflection from the authors**	[text]
**Notes**	[text]

Appendix 5: GRADE Quality assessment criteria *Journal of Clinical Epidemiology* 2011 64, 383-394DOI: (10.1016/j.jclinepi.2010.04.026) Copyright © 2011 Elsevier Inc. Terms and Conditions

<table>
<tr><th>Study Design</th><th>Quality of Evidence</th><th>Lower if</th><th>Higher if</th></tr>
<tr><td>Randomized trial →</td><td>High</td><td rowspan="4">Risk of bias
-1 Serious
-2 Very serious

Inconsistency
-1 Serious
-2 Very serious

Indirectness
-1 Serious
-2 Very serious

Imprecision
-1 Serious
-2 Very serious

Publication bias
-1 Likely
-2 Very likely</td><td rowspan="4">Large effect
+1 Large
+2 Very large

Dose response
+1 Evidence of a gradient

All plausible confounding
+1 Would reduce a demonstrated effect or

+1 Would suggest a spurious effect when results show no effect</td></tr>
<tr><td></td><td>Moderate</td></tr>
<tr><td>Observational study →</td><td>Low</td></tr>
<tr><td></td><td>Very low</td></tr>
</table>
