# Supplementary material for: Improving quality of surgical and anaesthesia care at hospital level in sub-Saharan Africa: a systematic review protocol of health system strengthening interventions
Source: BMJ Open. 2020 May 30;10(5):e036615. doi: 10.1136/bmjopen-2019-036615 (PMC7264698; doi:10.1136/bmjopen-2019-036615)
Supplement: Author's manuscript [file bmjopen-2019-036615.draft_revisions.pdf]

BMJ Open is committed to open peer review. As part of this commitment we make the peer review history of every article we publish publicly available.

When an article is published we post the peer reviewers' comments and the authors' responses online. We also post the versions of the paper that were used during peer review. These are the versions that the peer review comments apply to.

The versions of the paper that follow are the versions that were submitted during the peer review process. They are not the versions of record or the final published versions. They should not be cited or distributed as the published version of this manuscript.

BMJ Open is an open access journal and the full, final, typeset and author-corrected version of record of the manuscript is available on our site with no access controls, subscription charges or pay-per-view fees (http://bmjopen.bmj.com).

If you have any questions on BMJ Open's open peer review process please email info.bmjopen@bmj.com

BMJ Open

Improving quality of surgical and anaesthesia care at hospital level in Sub-Saharan Africa: a systematic review protocol of health system strengthening interventions.

Journal:	*BMJ Open*
Manuscript ID	bmjopen-2019-036615
Article Type:	Protocol
Date Submitted by the Author:	21-Dec-2019
Complete List of Authors:	Brima, Nataliya; King's College London, King's Centre for Global Health & Health Partnerships<br>Leather, Andy; King's Centre for Global Health and Health Partnerships<br>Davies, Justine ; Kings College London , Global Health
Keywords:	SURGERY, Quality in health care < HEALTH SERVICES ADMINISTRATION & MANAGEMENT, PUBLIC HEALTH

SCHOLARONE™
Manuscripts

I, the Submitting Author has the right to grant and does grant on behalf of all authors of the Work (as defined in the below author licence), an exclusive licence and/or a non-exclusive licence for contributions from authors who are: i) UK Crown employees; ii) where BMJ has agreed a CC-BY licence shall apply, and/or iii) in accordance with the terms applicable for US Federal Government officers or employees acting as part of their official duties; on a worldwide, perpetual, irrevocable, royalty-free basis to BMJ Publishing Group Ltd ("BMJ") its licensees and where the relevant Journal is co-owned by BMJ to the co-owners of the Journal, to publish the Work in this journal and any other BMJ products and to exploit all rights, as set out in our licence.

The Submitting Author accepts and understands that any supply made under these terms is made by BMJ to the Submitting Author unless you are acting as an employee on behalf of your employer or a postgraduate student of an affiliated institution which is paying any applicable article publishing charge ("APC") for Open Access articles. Where the Submitting Author wishes to make the Work available on an Open Access basis (and intends to pay the relevant APC), the terms of reuse of such Open Access shall be governed by a Creative Commons licence – details of these licences and which Creative Commons licence will apply to this Work are set out in our licence referred to above.

Other than as permitted in any relevant BMJ Author's Self Archiving Policies, I confirm this Work has not been accepted for publication elsewhere, is not being considered for publication elsewhere and does not duplicate material already published. I confirm all authors consent to publication of this Work and authorise the granting of this licence.

For peer review only

Systematic Review protocol

Improving quality of surgical and anaesthesia care at hospital level in Sub-Saharan Africa: a systematic review protocol of health system strengthening interventions.

PROSPERO Registration number: CRD42019125570

Nataliya Brima[1], Justine Davies[2], Andy Leather[1]

Corresponding author:

Mrs Nataliya Brima

[1]King's Centre for Global Health and Health Partnerships

School of Population Health and Environmental Sciences

Faculty of Life Sciences and Medicine

King's College London

Suite 2.13, Weston Education Centre

Cutcombe Road

London

SE5 9RJ

UK

nataliya.brima@kcl.ac.uk

+447930271138

Prof Justine Davies

[2]Institute for Applied Health Research, University of Birmingham, UK

J.Davies.6@bham.ac.uk

Mr Andy Leather

[1]King's Centre for Global Health and Health Partnerships, School of Population Health and Environmental Sciences, Faculty of Life Sciences and Medicine, King's College London, UK

andy.leather@kcl.ac.uk

Key words: surgical and anaesthesia care, improving quality of care, health system strengthening, Sub-Saharan Africa

For peer review only

Abstract

Introduction: Over 5 billion people in the world do not have access to safe, affordable surgical and anaesthesia care when needed. In order to improve health outcomes in patients with surgical conditions, both access to care and the quality of care needs to be improved. A recent commission on high quality health highlighted that poor-quality care is now a bigger barrier than non-utilisation of the health system for reducing mortality.

Aim: To carry out a systematic review to provide an evidence-based summary of hospital-based interventions associated with improved quality of surgical and anaesthesia care in Sub-Saharan African countries (SSACs).

Methods and Analysis: Three search strings: 1) surgery and anaesthesia, 2) quality improvement hospital-based interventions, 3) SSACs will be combined.

The following databases: EMBASE, Global Health, MEDLINE, CINAHL, Web of Science and Scopus will be searched. Further relevant studies will be identified from national and international health organisations and publications and reference lists of all selected full text articles.

The review will include all type of original articles in English. Article screening, data extraction and assessment of methodological quality will be done by two reviewers independently and any disputes will be resolved by a third reviewer or team consensus. The primary outcome is mortality. Secondary outcomes include major and minor complications, as well as clinical process outcomes and implementation outcomes. Descriptive statistics and outcomes will be summarised and discussed. For the primary outcome the methodological rigour will be assessed.

Ethics and Dissemination: The results will be published in a peer reviewed open access journal and presented at national and international conferences. As this is a review of secondary data no formal ethical approval is required.

Conclusion: The results will inform development of Quality Improvement Intervention (QII) in surgical and anaesthetic care in the hospital setting in SSACs.

Protocol PROSPERO registration number CRD42019125570

Strength and Limitations of this study

- A comprehensive search strategy for original articles and grey literature both published and unpublished was created and included.
- The inclusion of all fields of surgery in the search strategy will ensure that no health system strengthening interventions (HSSI) implemented within Sub-Saharan Africa (SSA) region are missed.
- It is possible that the included studies (and reported data) will be too few to address the objectives of this study.

For peer review only

- There is the potential that the data retrieved from different studies may not be applicable to every SSA country context due to differences in the healthcare system and therefore will need to be carefully adapted to the context of another country.

INTRODUCTION

The urgent need for surgical and anaesthesia care continues to be neglected, with over five billion people worldwide being deprived access to safe, affordable and timely surgical and anaesthesia care when needed [1]. SSACs are disproportionally affected where 93% of the population do not have access to basic surgical care [1]. Mortality and morbidity from treatable surgical diseases is high and continues to grow. In 2010, an estimated 32.9% of all lives lost were due to surgical burden of disease [2]. Untreated surgical diseases are among the top 15 causes of physical disability worldwide, with injuries accounting for 15% of all ill-health worldwide and 90% of deaths from injuries occurring in low- or middle- income countries (LMICs) [3], 85% of all SSACs are classifies as either low- or middle- income countries. The poorest countries account for 34·8% of the global population, yet annually, only 6% of surgical procedures worldwide take place in these countries [4] and SSA region has the greatest unmet need for surgical procedures [1]. Surgical patients in Africa are twice as likely to die after surgeries, despite being younger, lower-risk profile and developing less complications, compared to the global average [5].

Annually, 1.8 million deaths in LMICs could be surgically averted, with 1.4M treatable by basic surgical care delivered at first-level hospitals and 0.4M treatable by advanced surgical care delivered in specialised facilities [6]. This will require strong health systems with effective referral from community to primary care and onwards to secondary and tertiary hospital care [1] in order to address the barriers in accessing quality care. Recently, The Lancet Global Health Commission on High Quality Systems in SDG Era highlighted the low quality and performance of many LMIC health systems and placed emphasis on the prioritisation of high-quality care to reduce avoidable mortality and morbidity [7].

In this systematic review we will identify, critically appraise and synthesise the existing published evidence on health system interventions that have focused on quality surgical and anaesthesia care in hospitals in SSACs. This study will inform future efforts to improve the quality of hospital-based care for patients requiring surgical and anaesthesia care.

METHODS AND ANALYSIS

For peer review only

The systematic review will be conducted, and the data reported in accordance with the Preferred Reporting Items for Systematic Review and Meta-Analysis (PRISMA) statement [8]. This protocol was developed following the PRISMA Protocol (PRISMA-P) 2015 checklist [9, 10]. Amendments to the protocol are not anticipated but will be reported in the publication of the results, should they occur.

Aim

To provide an evidence-based summary of hospital-based interventions associated with improved quality of surgical and anaesthesia care in SSACs.

Objectives:

- To systematically identify studies that evaluate interventions aimed at improving quality of surgical and anaesthesia care at hospital level in SSACs.
- To capture, categorise and assess the study characteristics (setting and intervention).
- To capture, categorise and assess methodological rigour of studies' primary outcomes (clinical).
- To record whether process and implementation outcomes were assessed

Search strategy

The search string strategy was developed by the main author and checked by a research librarian for errors and omissions. During the development process the search was tested and retested to ensure optimal sensitivity and specificity and search terms refined accordingly. The search strategy was developed in MEDLINE (Appendix 1). The search terms used intended to cover a wide range of studies that evaluated interventions aimed at improving the quality of surgical and anaesthesia care at hospital level in SSACs. The three distinct search strings that were combined to identify these studies are: (1) surgical and anaesthesia care, (2) quality improvement hospital-based interventions, (3) SSACs.

Surgical and anaesthesia care will include all branches of surgery (cardiothoracic, ENT, general, neonatal, obstetric, ophthalmic, orthopaedic, paediatric and urology), including operative and non-operative aspects of care, on presumption that any branch of surgery may have relevant transferable interventions within a low resource setting. Trauma will be excluded as it will be covered in a separate systematic review.

For peer review only

For the purposes of this review HSSI is defined as any actions or strategies taken to improve the quality of health care delivery and patient outcomes. This will include studies that either strengthen the whole surgical system within a hospital or contribute to one aspect of surgical and anaesthesia care. The WHO Health System Framework will be used to categorise HSSIs [11].

Hospital-based interventions are defined as any intervention that is delivered within a tertiary/teaching/national or a secondary/district/regional care hospital environment, aimed at improving quality of care and that directly or indirectly involves care delivery to patients or staff. Both hospital-wide (hospital documentation, early warning scores, intensive care outreach) and individual departmental (emergency department, surgical wards, theatre, HDU, ITU, outpatient, radiology) interventions will be included.

The term SSACs will include all countries listed as Sub-Saharan Africa counties by the World Bank for the 2019 fiscal year [12].

To combine terms within each search string the Boolean operator 'OR' was used. To incorporate all variations of the same term, truncation and wildcards was employed. Medical Subject Headings (MeSH) terms and free text was also utilised. To focus the search on these required terms, phrase and adjacency searching, using proximity operator filters and limits was exploited. Any additional key terms identified during the literature search were added to the appropriate string to ensure it was maximally inclusive. The three strings were then combined using the Boolean operator 'AND'.

This search strategy will be adapted to EMBASE, Global Health, CINAHL, Web of Science databases. A reference list of all included articles will be hand-searched for further relevant studies.

To identify unpublished studies the Scopus literature databases will be searched. In addition, first 50 hits from Google search, documentation and reports of relevant national and international health organisations such as Comic Relief, DFID, Grand Challenges Canada, the Bill and Melinda Gates Foundation, HIVOS, World Vision, the Robert Wood Johnson foundation, UNISEF, GOALS will be searched.

The choice of information sources for this search were made based on the recommendation from the current literature, to ensure the optimal combination of databases and other search resources available needed to guaranty adequate and efficient coverage [13].

For peer review only

The search process will be recorded using a Database literature search log (Appendix 2).

Eligibility criteria

All eligible articles published in English will be included, from 2008 to 2019. The inclusion and exclusion criteria are illustrated in **Error! Reference source not found. Error! Reference source not found.**.

Table 1: Inclusion and Exclusion criteria

	**Include**	**Exclude**
Type of Article	All Peer-reviewed research articles<br><br>Non-research reports from national or international health organisations, dissertations/theses, books/book chapters, conference abstracts, and research in progress from grey literature	Unstructured reviews or overviews, theoretical papers, commentaries or opinion papers,<br><br>Case studies, audits, editorials/ letters/ comments, newspapers/ trade journals, literature reviews<br><br>Guidelines, strategies and policies from national or international health organisations
Type of conditions	Any surgical and anaesthesia care (operative or non-operative); type of presentation (elective or emergency); sub-speciality surgical or anaesthesia care (including peri-operative medicine and pain management).	Trauma/injury care<br><br>Studies on cosmetic and aesthetic surgical care and sports medicine
Type of population	General population<br><br>Population with specific surgical diseases or conditions<br><br>Adults, neonatal and children's surgery	Animals
Care setting	Hospital setting, SSACs	Studies that are not conducted in hospital-based settings.<br><br>Studies that took place outside of SSA
Type of design	Interventional studies[1]	Observational studies
Subject of study	Quality improvement of surgical	-Studies that did not assess

For peer review only

	care through the following areas:<br>-Service delivery<br>-Health Workforce<br>-Information<br>-Financing<br>-Leadership/Governance	outcomes<br>-Medical device production and new clinical technological devices<br>-Introduction of new procedures<br>-Medical products, vaccines and technologies

[1] Interventional studies are often prospective and are specifically tailored to evaluate direct impacts of treatment or preventive measures on disease [14].

Data extraction

All potential articles will be initially collated in Endnote X8 for de-duplication of results. All remaining studies will be loaded into Rayyan online opensource web application [15] and initially screened by title and abstract by a team of two reviewers independently, according to inclusion and exclusion criteria (Table 1) and by using the Systematic Review Abstract and Full Text Screening Criteria form (Appendix 3). All reviewers were appropriately trained in systematic review methods. If either of reviewers includes an abstract, it will be promoted to consideration for full text review. The full text review will be done by the first author (Nataliya Brima) and another reviewer independently, using inclusion and exclusion criteria. Data elements will be extracted into a predefined data extraction form (Appendix 4), which will be piloted before finalising the elements to be included. This includes information on authors, publication dates, country of intervention, health care setting, type of intervention, methodology or design, patient/intervention size, clinical/process/implementation outcomes and the impact of the intervention. The data extracted by two reviews will be compared and if any discrepancies arise, consensus will be sought among the study team (Figure 1).

For peer review only

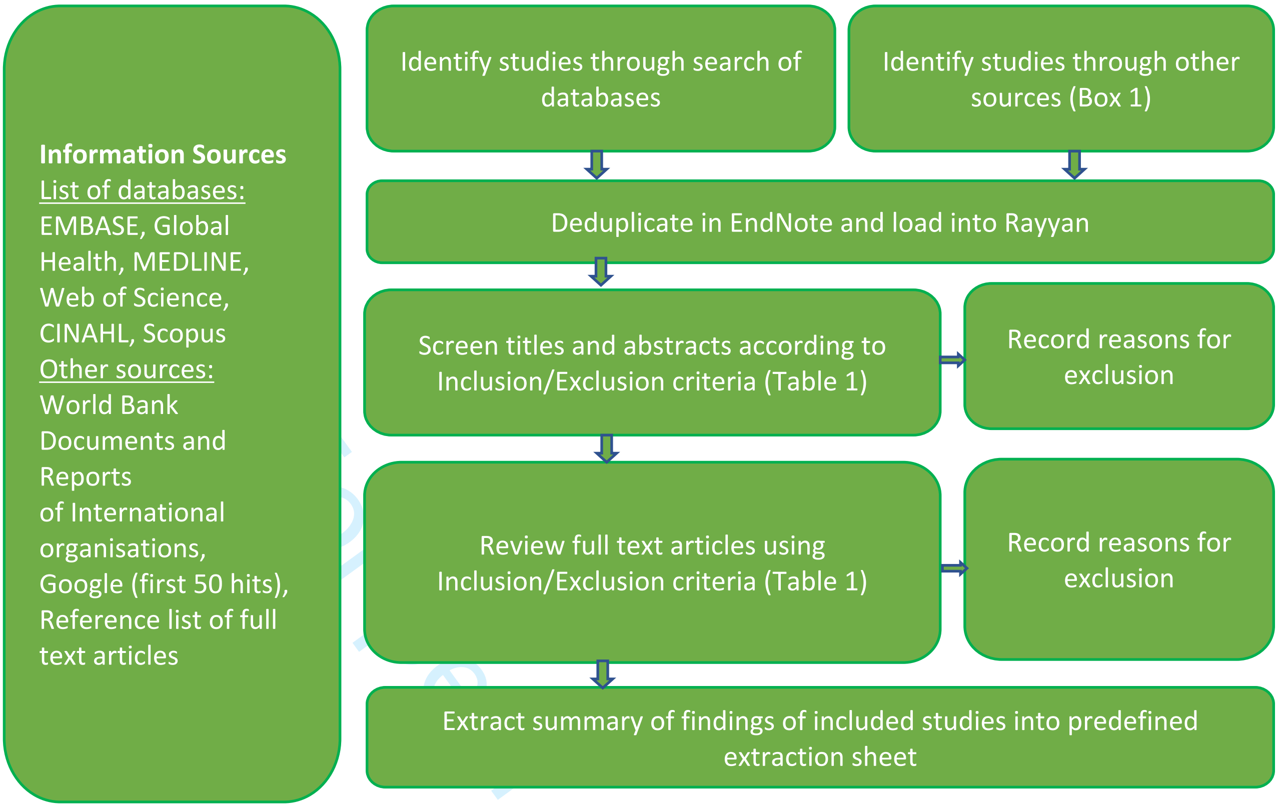

Figure 1: Data search and extraction flow diagram

Outcomes

Three type of outcomes- clinical, process and implementation will be collected. The clinical outcomes will be mortality (in-hospital perioperative mortality and 30-day post intervention mortality) and major and minor complications as defined by the Clavien-Dindo Classification (Table 2). Process outcomes will include all outcomes relevant to interventional studies included in this review (waiting times, length of hospital stay, blood availability, etc). Implementation outcomes will be determined using a ‘working taxonomy’ of 8 conceptually distinct implementation outcomes produced by Proctor et al [16] (Table 3).

Table 2: Clavien-Dindo Classification of Complications

Grade	Definition
I	Any deviation from the normal post-operative course without the need for pharmacological treatment or surgical, endoscopic and radiological interventions. Allowed therapeutic regimes are as follows: drugs as anti-emetics, anti-pyretics, analgesics, diuretics, electrolytes and

	physiotherapy. This grade also includes wound infections opened at the bedside.
II	Requiring pharmacological treatment with drugs other than such allowed for grade I complications. Blood transfusions and total parenteral nutrition are also included.
III	Requiring surgical, endoscopic, or radiological intervention.
IIIa	Intervention not under general anaesthesia.
IIIb	Intervention under general anaesthesia.
IV	Life-threatening complication requiring ICU management.
IVa	Single organ dysfunction (including dialysis).
IVb	Multi-organ dysfunction.
V	Death of a patient.

Table 3: Implementation Outcomes

	Implementation Outcome (Proctor et al)	Definition
1	Acceptability	Degree to which the intervention is perceived agreeable, acceptable or satisfactory
2	Adoption	Intention and willingness to apply the intervention
3	Appropriateness	Perceived relevance of the intervention to be relevant to the problem
4	Feasibility	Extent to which the intervention can be successfully applied
5	Fidelity	Extent to which the intervention is completed as originally intended
6	Implementation Cost	Intervention time and cost
7	Penetration	Spread into practice, e.g proportion of eligible patients who received the intervention
8	Sustainability	Extent to which a new intervention routinely is used in practice

Risk Of Bias

Risk of bias of individual studies and publication bias will be addressed by designing a thorough search strategy to find relevant studies. The search strategy will be assessed by checking the results against known articles which are relevant. To address publication bias the grey literature will be searched, as described above, to include all relevant studies which are not reported in peer-reviewed journals. The following type of publications will be included: dissertations/theses, books/book chapters, conference abstracts, and research in progress. Also, all potential full text articles will be screened by two reviewers who will independently assess the risk of bias as part of the Grading of Recommendations Assessment, Development, and Evaluation (GRADE) system [17] of rating quality of evidence and grading strength of recommendations in systematic reviews.

For peer review only

Disagreements between the reviews over the risk of bias level (serious or very serious) will be resolved by discussion with involvement of a third reviewer where necessary. Due to expected high heterogenicity of the data extracted, a meta-analysis will not be feasible.

Data synthesis

For all outcomes all items that will be collected using predefined collection form (Appendix 4) will be summarised and the methodological approach used in the studies will be identified. Descriptive statistics and narrative synthesis of data will be undertaken to appreciate the diversity of research approaches that are used for assessment of quality of surgical and anaesthesia care within hospital.

In addition, the main clinical outcomes of interventions will be assessed using GRADE criteria for rating quality of evidence and grading strength of recommendations in systematic reviews. [17]. All evidence, that used for rating the quality of evidence and include study design, risk of bias, imprecision, inconsistency, indirectness, and magnitude of effect, will be summarised in summary of findings table (Appendix 5). Based on this information quality of evidence will be rated and detailed information about the reason for the quality of evidence rating will be recorded. Outcomes will be categorised as strong or weak according to the quality of the supporting evidence.

ETHICS AND DISSEMINATION

This study is a part of a wider NIHR Global Health Research Unit on Health System Strengthening in Sub-Saharan Africa (ASSET) Sierra Leone project. The results will be communicated and disseminated during meetings with key stakeholders at national, district and community levels, that will be on-going throughout the project. The results will inform the development and implementation of a quality improvement intervention to improve quality of surgical and anaesthesia care in SSACs in general and in Sierra Leone in particular. The results of the review will be published in a peer reviewed journal and presented at national and international conferences. As this is a review of secondary data no formal ethical approval is required.

CONCLUSIONS

To our knowledge this is the first systematic literature review to synthesise the information on HSSIs to improve the quality of hospital-based surgical and anaesthesia care in SSACs. The findings from this systematic review will provide evidence to inform the development,

For peer review only

design and implementation of the HSSIs to improve the quality of health care for surgical patients at hospital level in SSACs. It will also provide valuable information for policymakers, healthcare managers and clinicians.

REFERENCES

1. Meara JG, Leather AJM, Hagander L, et al. Global Surgery 2030: Evidence and solutions for achieving health, welfare, and economic development. Lancet. 2015;386(9993):569-624. doi:10.1016/S0140-6736(15)60160-X. (Accessed 19th December 2019).
2. Shrime MG, Bickler SW, Alkire BC, Mock C. Global burden of surgical disease: an estimation from the provider perspective. Lancet Glob Health. 2015;3 Suppl2:S8-9. (Accessed 19th December 2019).
3. Gosselin RA, Spiegel DA, Coughlin R, Zirkle LG. Injuries: the neglected burden in developing countries. Bull World Health Organ. 2009;87(4):246-a. (Accessed 19th December 2019).
4. Weiser TG, Haynes AB, Molina G, et al. Estimate of the global volume of surgery in 2012: an assessment supporting improved health outcomes. Lancet 2015; 385 (Global Surgery special issue): S11 (Accessed 19th December 2019).
5. Biccard BM, Madiba TE, Kluyts HL, et al. African Surgical Outcomes Study (ASOS) investigators. (Accessed 19th December 2019).
6. Bickler SN, Weiser TG, Kassebaum N, et al. Editors In: Debas HT, Donkor P, Gawande A, Jamison DT, Kruk ME, Mock CN, editors. Source Essential Surgery: Disease Control Priorities, Third Edition (Volume 1). Washington (DC): The International Bank for Reconstruction and Development / The World Bank; 2015 Apr. Chapter 2. (Accessed 19th December 2019).
7. Kruk M E, Gage A D, Arsenault C, et al, High-quality health systems in the Sustainable Development Goals era: time for a revolution The Lancet Global Health, Volume 7, Issue 3, March 2019, Pages e303-e304 (Accessed 19th December 2019).
8. *Liberati A, Altman DG, Tetzlaff J, et al.* The PRISMA statement for reporting systematic reviews and meta-analyses of studies that evaluate healthcare interventions: explanation and elaboration.
BMJ. 2009 Jul 21; 339():b2700. (Accessed 19th December 2019).

9. Shamseer L, Moher D, Clarke M, et al. Preferred reporting items for systematic review and meta-analysis protocols (PRISMA-P) 2015: elaboration and explanation. BMJ 2015;349:g7647. (Accessed 19th December 2019).

10. Moher D, Liberati A, Tetzlaff J, et al. Preferred reporting items for systematic reviews and meta-analyses: the PRISMA statement. BMJ 2009;339: b2535. (Accessed 19th December 2019).

11. World Health Organization Everybody's Business: Strengthening Health Systems to Improve Health Outcomes: WHO's Framework for Action 2007 Geneva World health Organization (Accessed 19th December 2019).

12. World Bank. World Bank Country and Lending Groups. 2018. https://datahelpdesk.worldbank.org/knowledgebase/articles/906519-world-bank-country-and-lending-groups (Accessed 19th December 2019).

13. Bramer W, Rethlefsen M, Kleijnen J, et al. Optimal database combinations for literature searches in systematic reviews: a prospective exploratory study. Systematic reviews 2017; 6:245. (Accessed 19th December 2019).

14. Observational and interventional study design types; an overview. BioMed. 2014; 24(2):199-210 https://www.researchgate.net/publication/263475899_Observational_and_interventional_study_design_types_an_overview. (Accessed 19th December 2019).

15. Ouzzani, M, Hammady H, Fedorowicz Z, et al., Rayyan-a web and mobile app for systematic reviews. Syst Rev, 2016. 5(1): p. 210.

16. Proctor E et al. Outcomes for implementation research: conceptual distinctions, measurement challenges, and research agenda. Adm Policy Ment Health 2011; 38, 2:65-76 (Accessed 19th December 2019).

17. Guyatt 2011a. Guyatt G, Oxman AD, et al. GRADE guidelines: 1. Introduction—GRADE evidence profiles and summary of findings tables. Journal of Clinical Epidemiology 2011; 64: 383–394. (Accessed 19th December 2019).

For peer review only

Acknowledgments

This study is part of Health System Strengthening in Sub-Saharan Africa (ASSET) and supported by the King's Centrefor Global Health and Health Partnership. The authors thank the library services at King's College London, UK.

Author Statement:

NB, AL and JD conceived the idea for the study and designed the protocol. NB drafted the protocol and is the guarantor of the review. All authors provided feedback on the study design and review methods and contributed to the final manuscript.

Funding:

This systematic review is supported by the NIHR Global Health Research Unit on Health Systems Strengthening in Sub-Saharan Africa, King's College London (GHRU 16/136/54) The funding body had no input into the study design.

Competing interests:
The authors declare no competing interests.

Word count: 1815

For peer review only

Appendix1: Search strategies for MEDLINE

1) Surgical or anaesthesia ;2) quality improvement hospital level interventions;3) Sub-Saharan African countries (SSACs)

Search String 1	Search String 2	Search String 3
surg* OR surgic* OR an?esthes* OR operat* OR pre?operat* OR post?operat* OR obstetr*<br>OR laparotomy OR ambulatory surgical procedures/ or exp laparotomy/ or exp minor surgical procedures/ or obstetric surgical procedures/ or exp perioperative care/ or perioperative exp period/ OR exp General Surgery/ OR exp Anesthetics, General/ or exp Anesthetics, Inhalation/ or exp Anesthetics, Combined/ or exp Anesthetics/ or exp Anesthetics, Local/ or	Hospital* OR in?patient* OR ward* OR (intensive adj1 care*).mp OR exp Hospitals/ OR exp Intensive Care Units, Pediatric/ or exp Hospital Units/ or exp Mobile Health Units/ or exp Coronary Care Units/ or exp Intensive Care Units/ or exp Colony-Forming Units Assay/ or exp Burn Units/ or exp Intensive Care Units, Neonatal/ OR exp inpatient/ OR (Operat* adj2 (room* or theatre)).mp<br>AND (any of the following)<br>intersectoral collaboration/ or efficiency, organizational/ or "facilities and services utilization"/ or hospital information systems/ or hospital-patient relations/ or database management systems/ or ambulatory care information systems/ or medical order entry systems/ or operating room information systems/ or point-of-care systems/ or "personnel staffing and scheduling information systems"/ or medication systems, hospital/ or patient identification systems/ or health smart cards/ or radio frequency identification device/ or employee discipline/ or employee incentive plans/ or employee performance appraisal/ or staff development/ or time management/<br>advance directive adherence/ or exp clinical competence/ or exp guideline adherence/ or exp patient reported outcome measures/ or exp alert fatigue, health personnel/ or exp benchmarking/ or exp clinical audit/ or exp credentialing/ or exp "facility regulation and control"/ or exp guidelines as topic/ or exp Quality Control/ or exp "Quality of Life"/ or exp Quality-Adjusted Life Years/<br>OR "Reminder Systems"/ or "Total Quality Management"/ or exp "Patient Education"/ or "Health Education"/ or exp "Consumer Health Information"/ or "Critical Pathways"/ or "Education, Medical, Continuing"/ or exp "Inservice Training"/ or "Guideline Adherence"/ or "Clinical Competence"/ or "Peer Review"/ or exp "Medical Audit"/ OR OR patient education.mp OR critical pathway*.mp OR clinical pathway*.mp OR care pathway*.mp OR (continuing adj2 education).mp OR (inservice or in service).mp OR (staff adj3 train*).mp OR audit* or feedback* OR exp Medical Record Linkage/ OR exp Patient Safety/ or exp Safety Management/ OR "task performance and analysis"/ or "time and motion studies"/ or work simplification/ OR<br>"patient risk management".mp OR “clinical documentation” OR ”nursing documentation” OR (clinical adj2 record*) OR (patient* adj2 record*) OR (hospital adj2 documentation) OR (nursing process) OR (Patient adj2 care plan*)	sub?Saharan Africa*.mp OR Angola or Benin or Bolivia or "Burkina Faso" or Burundi or "Cabo Verde" or Cameroon or "Central African Republic" or Chad or Comoros or Congo or "Democratic Republic of the Congo" or DRC or "Republic of the Congo" or "Cote d'Ivoire" or "Ivory Coast" or "Equatorial Guinea" or Eritrea or Eswatini or Ethiopia or Gabon or Gambia or Ghana or Guinea or "Guinea?Bissau" or Kenya or Lesotho or Liberia or Madagascar or Malawi or Mali or Mauritania or Mauritius or Mozambique or Namibia or Niger or Nigeria or Rwanda or "Sao

For peer review only

exp Anesthetics, Intravenous/ or exp Anesthetics, Dissociative/ OR exp Obstetrics/	OR (nurs* adj2 documentation adj2 framework*) OR<br>OR (audit adj3 feedback).mp OR (safety checklist* or safety check list*).mp, (clinical adj2 record*).mp, (patient* adj2 record*).mp, (clinical adj2 documentation).mp, (data adj2 qualit*).mp, (hospital adj2 documentation).m<br>AND<br>exp Quality Improvement/ OR "Outcome and Process Assessment"/ or "Outcome Assessment"/ or "Process Assessment"/ or "Quality of Health Care"/ or "Quality Assurance"/ or "Quality Improvement"/ or "Quality Indicators"/ or "Management Quality Circles" OR (outcome* adj3 assessment*).mp OR (process* adj3 assessment*).mp OR quality assurance.mp OR quality improvement.mp OR (improvement adj intervention*).mp OR (improvement adj program*).mp OR (improvement adj initiative*).mp OR (process* adj improvement).mp OR quality indicator*.mp OR quality circle*.mp OR (total quality management or six sigma*).mp OR (program* adj3 effectiveness).mp OR (program* adj3 evaluation*).mp OR "Program Evaluation"/ or quality* or safet*	Tome and Principe" or Senegal or "Sierra Leone" or Seychelles or Somalia or "South Africa" or "South Sudan" or Sudan or Tanzania or Togo or Uganda or Zambia or Zimbabwe

*Include USA spelling, plurals, and synonym

For peer review only

Appendix2: Database literature search log.

Date	Database Name	Search Terms	Search Limits	Results	Comments

Apendix3: Systematic Review Abstract and Full Text Screening Criteria

Topic	Criteria	Yes	Unclear	No
Type of article	Is this a Peer-reviewed research articles			
Health Intervention care setting (**HICS**)	Is this intervention in hospital-based setting			
Type of condition	1.Does this study relate to any surgical and anaesthesia care presentation			
	2.Does this study relate to trauma/injury care, cosmetic surgery or sport medicine?			
Outcome	Does this study report Outcomes-Clinicla, Process, Implementation			
Subject of study	Does this study about Medical production, vaccines and technologies			

Instruction

- Include Abstract and Find Full Text if Type of article (Yes) AND HICS (Yes) AND Type of conditions 1 (Yes) AND Type of conditions 2 (No/Unclear) AND Outcome (Yes) and Subject Study (No)
- Exclude Abstract and Full text if Type of article (Unclear/No) OR HICS (Unclear/No) OR Type of conditions 1 (Unclear/No) OR Type of conditions 2 (Yes) OR Outcome (No) OR Subject Study (Yes)

For peer review only

Appendix 4: Systematic Review Data Extraction Form

Variable	Response
**Reference**	[text]
**Year**	[number]
**Language**	[text]
**Location(s)**	[text]
**Type of publication**	[Grey (1) /Postgrad Dissertation (2)/Published- peer reviewed (3)/Non-research report (4)]
**Journal**	[text]
**Journal Field**	[text]
**Name of low and middle income country**	[text]
**Brief description of study**	[text]
**Surgical and anaesthesia care Intervention**	[yes/no]
**Integrated into routine health services**	[yes/no]
**Health care setting**	[Tertiary (1)/ Secondary Hospital (2)/ Level-hospital department (triage, surgical theatre) (3)]
**Study Methodology**	[Qualitative (1)/quantitative (2)]/ mixed (3)
**Study design**	[RCT, Observational, cohort, quazi-experimental, etc)]
**Describes Evaluation of the intervention**	[retrospective/prospective/neither]
**Type of health intervention**	[Service delivery (1)/Health Workforce (2)/Information (3)/ Financing (4)/Leadership/Governance (5)]
**Population size/Sample size**	
**Primary clinical outcome**	[e.g mortality, length of stay, SSI]
**Result of Primary Outcome**	[test]
**Secondary Clinical outcome collected and assessed**	[major or minor complications]
**Process outcomes collected and assessed**	[text]
**Implementation outcome collected and assessed**	[text]
**Describes design of intervention**	[text] e.g System thinking, TOC, LF
**Describes Implementation of intervention**	[text] e.g Fidelity, Adoption
**Type of impact**	[text]
**Detail on statistical analysis**	[text]
**Potential Biases or concerns**	[text]
**Cost/source of funding for intervention**	
**Other points or key lessons for reflection from the authors**	[text]
**Notes**	[text]

For peer review only

Appendix 5: GRADE Quality assessment criteria *Journal of Clinical Epidemiology* 2011 64, 383-394DOI: (10.1016/j.jclinepi.2010.04.026) Copyright © 2011 Elsevier Inc. Terms and Conditions

Study Design	Quality of Evidence	Lower if	Higher if
Randomized trial →	High	Risk of bias<br>-1 Serious<br>-2 Very serious	Large effect<br>+1 Large<br>+2 Very large
	Moderate	Inconsistency<br>-1 Serious<br>-2 Very serious	Dose response<br>+1 Evidence of a gradient
Observational study →	Low	Indirectness<br>-1 Serious<br>-2 Very serious<br><br>Imprecision<br>-1 Serious<br>-2 Very serious	All plausible confounding<br>+1 Would reduce a demonstrated effect or<br><br>+1 Would suggest a spurious effect when results show no effect
	Very low	Publication bias<br>-1 Likely<br>-2 Very likely	

review only

Supplementary File 1

Improving quality of surgical and anaesthesia care at hospital level in SSACs: a systematic review protocol of health system strengthening interventions.

PRISMA-P (Preferred Reporting Items for Systematic review and Meta-Analysis Protocols) 2015 checklist: recommended items to address in a systematic review protocol*

Section and topic	Item No	Checklist item	Section where each item is addressed in the protocol
**ADMINISTRATIVE INFORMATION**			
Title:			
Identification	1a	Identify the report as a protocol of a systematic review	Title page
Update	1b	If the protocol is for an update of a previous systematic review, identify as such	N/A
Registration	2	If registered, provide the name of the registry (such as PROSPERO) and registration number	Title page
Authors:			
Contact	3a	Provide name, institutional affiliation, e-mail address of all protocol authors; provide physical mailing address of corresponding author	Title page
Contributions	3b	Describe contributions of protocol authors and identify the guarantor of the review	Contributions
Amendments	4	If the protocol represents an amendment of a previously completed or published protocol, identify as such and list changes; otherwise, state plan for documenting important protocol amendments	Methods and Analysis
Support:			
Sources	5a	Indicate sources of financial or other support for the review	Funding
Sponsor	5b	Provide name for the review funder and/or sponsor	Funding
Role of sponsor or funder	5c	Describe roles of funder(s), sponsor(s), and/or institution(s), if any, in developing the protocol	Funding
**INTRODUCTION**			
Rationale	6	Describe the rationale for the review in the context of what is already known	Introduction
Objectives	7	Provide an explicit statement of the question(s) the review will address with reference to participants, interventions, comparators, and outcomes (PICO)	Aim and Objectives
**METHODS**			
Eligibility criteria	8	Specify the study characteristics (such as PICO, study design, setting, time frame) and report characteristics (such	Search strategy, Eligibility

For peer review only

		as years considered, language, publication status) to be used as criteria for eligibility for the review	criteria, Table 1, Appendix 3
Information sources	9	Describe all intended information sources (such as electronic databases, contact with study authors, trial registers or other grey literature sources) with planned dates of coverage	Search strategy, Figure 1, Appendix 2
Search strategy	10	Present draft of search strategy to be used for at least one electronic database, including planned limits, such that it could be repeated	Appendix 1
Study records:			
Data management	11a	Describe the mechanism(s) that will be used to manage records and data throughout the review	Data extraction
Selection process	11b	State the process that will be used for selecting studies (such as two independent reviewers) through each phase of the review (that is, screening, eligibility and inclusion in meta-analysis)	Data extraction
Data collection process	11c	Describe planned method of extracting data from reports (such as piloting forms, done independently, in duplicate), any processes for obtaining and confirming data from investigators	Data extraction
Data items	12	List and define all variables for which data will be sought (such as PICO items, funding sources), any pre-planned data assumptions and simplifications	Appendix 4 and Data Synthesis
Outcomes and prioritization	13	List and define all outcomes for which data will be sought, including prioritization of main and additional outcomes, with rationale	Outcomes
Risk of bias in individual studies	14	Describe anticipated methods for assessing risk of bias of individual studies, including whether this will be done at the outcome or study level, or both; state how this information will be used in data synthesis	Risk of bias
Data synthesis	15a	Describe criteria under which study data will be quantitatively synthesised	Data Synthesis
	15b	If data are appropriate for quantitative synthesis, describe planned summary measures, methods of handling data and methods of combining data from studies, including any planned exploration of consistency (such as I^2, Kendall's τ)	Appendix 5
	15c	Describe any proposed additional analyses (such as sensitivity or subgroup analyses, meta-regression)	Data Synthesis
	15d	If quantitative synthesis is not appropriate, describe the type of summary planned	Data Synthesis
Meta-bias(es)	16	Specify any planned assessment of meta-bias(es) (such as publication bias across studies, selective reporting within studies)	Data Synthesis
Confidence in cumulative evidence	17	Describe how the strength of the body of evidence will be assessed (such as GRADE)	Data Synthesis

*** It is strongly recommended that this checklist be read in conjunction with the PRISMA-P Explanation and Elaboration (cite when available) for important clarification on the items. Amendments to a review protocol should be tracked and dated.** **The copyright for PRISMA-P (including checklist) is held by the PRISMA-P Group and is distributed under a Creative Commons Attribution Licence 4.0.**

From: Shamseer L, Moher D, Clarke M, Ghersi D, Liberati A, Petticrew M, Shekelle P, Stewart L, PRISMA-P Group. Preferred reporting items for systematic review and meta-analysis protocols (PRISMA-P) 2015: elaboration and explanation. BMJ. 2015 Jan 2;349(jan02 1):g7647.

For peer review only

Improving quality of surgical and anaesthesia care at hospital level in Sub-Saharan Africa: a systematic review protocol of health system strengthening interventions.

Journal:	*BMJ Open*
Manuscript ID	bmjopen-2019-036615.R1
Article Type:	Protocol
Date Submitted by the Author:	02-Mar-2020
Complete List of Authors:	Brima, Nataliya; King's College London, King’s Centre for Global Health & Health Partnerships<br>Davies, Justine ; Kings College London , Global Health<br>Leather, Andy; King's Centre for Global Health and Health Partnerships
**Primary Subject Heading**:	Surgery
Secondary Subject Heading:	Surgery
Keywords:	Quality in health care < HEALTH SERVICES ADMINISTRATION & MANAGEMENT, PUBLIC HEALTH, SURGERY

I, the Submitting Author has the right to grant and does grant on behalf of all authors of the Work (as defined in the below author licence), an exclusive licence and/or a non-exclusive licence for contributions from authors who are: i) UK Crown employees; ii) where BMJ has agreed a CC-BY licence shall apply, and/or iii) in accordance with the terms applicable for US Federal Government officers or employees acting as part of their official duties; on a worldwide, perpetual, irrevocable, royalty-free basis to BMJ Publishing Group Ltd ("BMJ") its licensees and where the relevant Journal is co-owned by BMJ to the co-owners of the Journal, to publish the Work in this journal and any other BMJ products and to exploit all rights, as set out in our licence.

The Submitting Author accepts and understands that any supply made under these terms is made by BMJ to the Submitting Author unless you are acting as an employee on behalf of your employer or a postgraduate student of an affiliated institution which is paying any applicable article publishing charge ("APC") for Open Access articles. Where the Submitting Author wishes to make the Work available on an Open Access basis (and intends to pay the relevant APC), the terms of reuse of such Open Access shall be governed by a Creative Commons licence – details of these licences and which Creative Commons licence will apply to this Work are set out in our licence referred to above.

Other than as permitted in any relevant BMJ Author's Self Archiving Policies, I confirm this Work has not been accepted for publication elsewhere, is not being considered for publication elsewhere and does not duplicate material already published. I confirm all authors consent to publication of this Work and authorise the granting of this licence.

For peer review only

Systematic Review protocol

Improving quality of surgical and anaesthesia care at hospital level in Sub-Saharan Africa: a systematic review protocol of health system strengthening interventions.

PROSPERO Registration number: CRD42019125570

Nataliya Brima[1], Justine Davies[2], Andy Leather[1]

Corresponding author:

Mrs Nataliya Brima
[1]King's Centre for Global Health and Health Partnerships
School of Population Health and Environmental Sciences
Faculty of Life Sciences and Medicine
King's College London
Suite 2.13, Weston Education Centre
Cutcombe Road
London
SE5 9RJ
UK
nataliya.brima@kcl.ac.uk
+447930271138

Prof Justine Davies
[2]Institute for Applied Health Research, University of Birmingham, UK
J.Davies.6@bham.ac.uk

Mr Andy Leather
[1]King's Centre for Global Health and Health Partnerships, School of Population Health and Environmental Sciences, Faculty of Life Sciences and Medicine, King's College London, UK
andy.leather@kcl.ac.uk

Key words: surgical and anaesthesia care, improving quality of care, health system strengthening, Sub-Saharan Africa

For peer review only

Abstract

Introduction: Over 5 billion people in the world do not have access to safe, affordable surgical and anaesthesia care when needed. In order to improve health outcomes in patients with surgical conditions, both access to care and the quality of care needs to be improved. A recent commission on high quality health highlighted that poor-quality care is now a bigger barrier than non-utilisation of the health system for reducing mortality.

Aim: To carry out a systematic review to provide an evidence-based summary of hospital-based interventions associated with improved quality of surgical and anaesthesia care in Sub-Saharan African countries (SSACs).

Methods and Analysis: Three search strings: 1) surgery and anaesthesia, 2) quality improvement hospital-based interventions, 3) SSACs will be combined.

The following databases: EMBASE, Global Health, MEDLINE, CINAHL, Web of Science and Scopus will be searched. Further relevant studies will be identified from national and international health organisations and publications and reference lists of all selected full text articles.

The review will include all type of original articles in English. Article screening, data extraction and assessment of methodological quality will be done by two reviewers independently and any disputes will be resolved by a third reviewer or team consensus. Three types of outcomes will be collected including clinical, process and implementation outcomes. The primary outcome will be mortality. Secondary outcomes will include other clinical outcomes (major and minor complications), as well as process and implementation outcomes. Descriptive statistics and outcomes will be summarised and discussed. For the primary outcome the methodological rigour will be assessed.

Ethics and Dissemination: The results will be published in a peer reviewed open access journal and presented at national and international conferences. As this is a review of secondary data no formal ethical approval is required.

Conclusion: The results will inform development of Quality Improvement Intervention (QII) in surgical and anaesthetic care in the hospital setting in SSACs.

Protocol PROSPERO registration number CRD42019125570

Strengths and Limitations of this study

- A comprehensive search strategy for original articles and grey literature both published and unpublished was created and included.
- The inclusion of all fields of surgery in the search strategy will ensure that no health system strengthening interventions (HSSI) implemented within Sub-Saharan Africa (SSA) region are missed.

For peer review only - http://bmjopen.bmj.com/site/about/guidelines.xhtml

For peer review only

- It is possible that the included studies (and reported data) will be too few to address the objectives of this study.
- There is the potential that the data retrieved from different studies may not be applicable to every SSA country context due to differences in the healthcare system and therefore will need to be carefully adapted to the context of another country.
- Although every effort will be made to address the publication bias we acknowledge that unsuccessful quality improvement efforts and unwanted side-effects are rarely published. Therefore, this review may not reflect the real current experience of failed attempts in the field of quality improvement of surgical care.

INTRODUCTION

The urgent need for surgical and anaesthesia care continues to be neglected, with over five billion people worldwide being deprived access to safe, affordable and timely surgical and anaesthesia care when needed [1]. SSACs are disproportionally affected where 93% of the population do not have access to basic surgical care [1]. Mortality and morbidity from treatable surgical diseases is high and continues to grow. In 2010, an estimated 32.9% of all lives lost were due to surgical burden of disease [2]. Untreated surgical diseases are among the top 15 causes of physical disability worldwide, with injuries accounting for 15% of all ill-health worldwide and 90% of deaths from injuries occurring in low- or middle- income countries (LMICs) [3], 85% of all SSACs are classified as either low- or middle- income countries. The poorest countries account for 34·8% of the global population, yet annually, only 6% of surgical procedures worldwide take place in these countries [4] and SSA region has the greatest unmet need for surgical procedures [1]. Surgical patients in Africa are twice as likely to die after surgeries, despite being younger, lower-risk profile and developing fewer complications, compared to the global average [5].

Annually, 1.8 million deaths in LMICs could be surgically averted, with 1.4M treatable by basic surgical care delivered at first-level hospitals and 0.4M treatable by advanced surgical care delivered in specialised facilities [6]. This will require strong health systems with effective referral from community to primary care and onwards to secondary and tertiary hospital care [1] in order to address the barriers in accessing quality care. Recently, the Lancet Global Health Commission on High Quality Health Systems in SDG Era highlighted the low quality and performance of many LMIC health systems and placed emphasis on the prioritisation of high-quality care to reduce avoidable mortality and morbidity [7].

In this systematic review we will identify, critically appraise and synthesise the existing published evidence on health system interventions that have focused on quality surgical and

For peer review only - http://bmjopen.bmj.com/site/about/guidelines.xhtml

anaesthesia care in hospitals in SSACs. This study will inform future efforts to improve the quality of hospital-based care for patients requiring surgical and anaesthesia care.

METHODS AND ANALYSIS

The systematic review will be conducted, and the data reported in accordance with the Preferred Reporting Items for Systematic Review and Meta-Analysis (PRISMA) statement [8]. This protocol was developed following the PRISMA Protocol (PRISMA-P) 2015 checklist [9, 10], (Supplementary File 1). Amendments to the protocol are not anticipated but will be reported in the publication of the results, should they occur.

Aim

To provide an evidence-based summary of hospital-based interventions associated with improved quality of surgical and anaesthesia care in SSACs.

Objectives:

- To systematically identify studies that evaluate interventions aimed at improving quality of surgical and anaesthesia care at hospital level in SSACs.
- To capture, categorise and assess the study characteristics (setting and intervention).
- To capture and categorise all outcomes reported under the following categories – clinical, process, implementation or other
- To assess methodological rigour of studies with mortality as a primary outcome (which is defined as mortality within 30 days defined for this review).
- To record whether process and implementation outcomes were assessed

Search strategy

The search string strategy was developed by the main author and checked by a research librarian for errors and omissions. During the development process the search was tested and retested to ensure optimal sensitivity and specificity and search terms refined accordingly. The search strategy was developed in MEDLINE (Appendix 1). The search terms used intended to cover a wide range of studies that evaluated interventions aimed at improving the quality of surgical and anaesthesia care at hospital level in SSACs. The three distinct search strings that were combined to identify these studies are: (1) surgical and anaesthesia care, (2) quality improvement hospital-based interventions, (3) SSACs.

For peer review only - http://bmjopen.bmj.com/site/about/guidelines.xhtml

Surgical and anaesthesia care will include all branches of surgery (cardiothoracic, ENT, general, neonatal, obstetric, ophthalmic, orthopaedic, paediatric and urology), including operative and non-operative aspects of care, on presumption that any branch of surgery may have relevant transferable interventions within a low resource setting. For the purpose of this review only studies that explicitly refer to a surgical procedure or a surgical patient will be included. Trauma (both explicitly focusing or included as part of the intervention) will be excluded, as it will be covered in a separate systematic review.

For the purposes of this review HSSI is defined as any actions or strategies taken to improve the quality of health care delivery and patient outcomes. This will include studies that either strengthen the whole surgical system within a hospital or contribute to one aspect of surgical and anaesthesia care. The WHO Health System Framework will be used to categorise HSSIs [11].

Hospital-based interventions are defined as any intervention that is delivered within a tertiary/teaching/national or a secondary/district/regional care hospital environment, aimed at improving quality of care, that directly or indirectly involves care delivery to patients or by staff. Both hospital-wide (hospital documentation, early warning scores, intensive care outreach) and individual departmental (emergency department, surgical wards, theatre, HDU, ITU, outpatient, radiology) interventions will be included.

The term SSACs will include all countries listed as Sub-Saharan Africa counties by the World Bank for the 2019 fiscal year [12].

To combine terms within each search string the Boolean operator 'OR' was used. To incorporate all variations of the same term, truncation and wildcards was employed. Medical Subject Headings (MeSH) terms and free text was also utilised. To focus the search on these required terms, phrase and adjacency searching, using proximity operator filters and limits was exploited. Any additional key terms identified during the literature search were added to the appropriate string to ensure it was maximally inclusive. The three strings were then combined using the Boolean operator 'AND'.

This search strategy will be adapted to EMBASE, Global Health, CINAHL, Web of Science databases. A reference list of all included articles will be hand-searched for further relevant studies.

To identify unpublished studies the Scopus literature databases will be searched. In addition, first 50 hits from Google search, documentation and reports of relevant national

For peer review only - http://bmjopen.bmj.com/site/about/guidelines.xhtml

and international health organisations such as Comic Relief, DFID, Grand Challenges Canada, the Bill and Melinda Gates Foundation, HIVOS, World Vision, the Robert Wood Johnson foundation, UNISEF, GOALS will be searched.

The choice of information sources for this search were made based on the recommendation from the current literature, to ensure the optimal combination of databases and other search resources available needed to guaranty adequate and efficient coverage [13].

The search process will be recorded using a Database literature search log (Appendix 2).

Eligibility criteria

All eligible articles published in English will be included, from 2008 to 2019. The inclusion and exclusion criteria are illustrated in **Error! Reference source not found. Error! Reference source not found.**.

Table 1: Inclusion and Exclusion criteria

	**Include**	**Exclude**
Type of Article	All Peer-reviewed research articles<br><br>Non-research reports from national or international health organisations, dissertations/theses, books/book chapters, conference abstracts, and research in progress from grey literature	Unstructured reviews or overviews,<br>theoretical papers,<br>commentaries or opinion papers,<br><br>Case studies,<br>audits,<br>editorials/ letters/ comments,<br>newspapers/ trade journals,<br>literature reviews<br><br>Guidelines, strategies and policies from national or international health organisations
Type of conditions	Any surgical and anaesthesia care (operative or non-operative); type of presentation (elective or emergency); sub-speciality surgical or anaesthesia care (including peri-operative medicine and pain management).	Trauma/injury care<br><br>Studies on cosmetic and aesthetic surgical care and sports medicine
Type of population	General population<br><br>Population with specific surgical diseases or conditions	Animals

For peer review only - http://bmjopen.bmj.com/site/about/guidelines.xhtml

	Adults, neonatal and children's surgery	
Care setting	Hospital setting, SSACs	Studies that are not conducted in hospital-based settings.<br><br>Studies that took place outside of SSA
Type of design	Interventional studies[1]	Observational studies
Subject of study	Quality improvement of surgical care through the following areas:<br>-Service delivery<br>-Health Workforce<br>-Information<br>-Financing<br>-Leadership/Governance	-Studies that did not assess outcomes.<br>-Medical device production and new clinical technological devices<br>-Introduction of new procedures<br>-Medical products, vaccines and technologies

[1] Interventional studies are often prospective and are specifically tailored to evaluate direct impacts of treatment or preventive measures on disease [14].

For peer review only

Data extraction

All potential articles will be initially collated in Endnote X8 for de-duplication of results. All remaining studies will be loaded into Rayyan online opensource web application [15] and initially screened by title and abstract by a team of two reviewers independently, according to inclusion and exclusion criteria (Table 1) and by using the Systematic Review Abstract and Full Text Screening Criteria form (Appendix 3). All reviewers were appropriately trained in systematic review methods. If either of reviewers includes an abstract, it will be promoted to consideration for full text review. The full text review will be done by the first author (Nataliya Brima) and another reviewer independently, using inclusion and exclusion criteria. Data elements will be extracted into a predefined data extraction form (Appendix 4), which will be piloted before finalising the elements to be included. This includes information on authors, publication dates, country of intervention, health care setting, type of intervention, methodology or design, patient/intervention size, clinical/process/implementation/other outcomes and the impact of the intervention. The data extracted by two reviews will be compared and if any discrepancies arise, consensus will be sought among the study team (Figure 1).

Outcomes

Three type of outcomes- clinical, process and implementation will be collected. The primary outcome for this review will be - mortality (in-hospital perioperative mortality and post intervention mortality within 30 days). All other outcomes will be secondary: clinical outcome such as major and minor complications as defined by the Clavien-Dindo Classification (Table 2) and any other clinical outcomes identified during the course of reviewing the manuscripts. Process outcomes will include all outcomes relevant to interventional studies included in this review (waiting times, length of hospital stay, blood availability, etc). Implementation outcomes will be determined using a 'working taxonomy' of 8 conceptually distinct implementation outcomes produced by Proctor et al [16] (Table 3). If any other outcomes are reported that cannot fit into the three categories above, they will be recorded under the heading 'Other outcomes', described, and categorised, if possible, after discussion amongst authors.

Table 2: Clavien-Dindo Classification of Complications

Grade	Definition
I	Any deviation from the normal post-operative course without the need for pharmacological treatment or surgical, endoscopic and radiological interventions. Allowed therapeutic regimes are as follows: drugs as anti-emetics, anti-pyretics, analgesics, diuretics, electrolytes and physiotherapy. This grade also includes wound infections opened at the bedside.
II	Requiring pharmacological treatment with drugs other than such allowed for grade I complications. Blood transfusions and total parenteral nutrition are also included.
III	Requiring surgical, endoscopic, or radiological intervention.
IIIa	Intervention not under general anaesthesia.
IIIb	Intervention under general anaesthesia.
IV	Life-threatening complication requiring ICU management.
IVa	Single organ dysfunction (including dialysis).
IVb	Multi-organ dysfunction.
V	Death of a patient.

Table 3: Implementation Outcomes

	Implementation Outcome (Proctor et al)	Definition
1	Acceptability	Degree to which the intervention is perceived agreeable, acceptable or satisfactory
2	Adoption	Intention and willingness to apply the intervention
3	Appropriateness	Perceived relevance of the intervention to be relevant to the problem
4	Feasibility	Extent to which the intervention can be successfully applied
5	Fidelity	Extent to which the intervention is completed as originally intended
6	Implementation Cost	Intervention time and cost

For peer review only - http://bmjopen.bmj.com/site/about/guidelines.xhtml

7	Penetration	Spread into practice, e.g proportion of eligible patients who received the intervention
8	Sustainability	Extent to which a new intervention routinely is used in practice

Risk Of Bias

Risk of bias of individual studies and publication bias will be addressed by designing a thorough search strategy to find relevant studies. The search strategy will be assessed by checking the results against known articles which are relevant. To address publication bias the grey literature will be searched, as described above, to include all relevant studies which are not reported in peer-reviewed journals. The following type of publications will be included: dissertations/theses, books/book chapters, conference abstracts, and research in progress. Also, all potential full text articles will be screened by two reviewers. For studies that reported their main outcome as mortality the reviewers will independently assess the risk of bias as part of the Grading of Recommendations Assessment, Development, and Evaluation (GRADE) system [17] of rating quality of evidence and grading strength of recommendations in systematic reviews.
Disagreements between the reviews over the risk of bias level (serious or very serious) will be resolved by discussion with involvement of a third reviewer where necessary. Due to expected high heterogenicity of the data extracted, a meta-analysis will not be feasible.

Data synthesis

For all outcomes all items that will be collected using predefined collection form (Appendix 4) will be summarised and the methodological approach used in the studies will be identified. Descriptive statistics and narrative synthesis of data will be undertaken to appreciate the diversity of research approaches that are used for assessment of quality of surgical and anaesthesia care within hospital. Those studies that were collected under the heading 'other', we will look for common themes and then compare these themes to our three defined categories of outcomes.

In addition, the studies reporting mortality as the main outcome of the interventions will be assessed using GRADE criteria for rating quality of evidence and grading strength of recommendations in systematic reviews. [17]. All evidence, that used for rating the quality of evidence and include study design, risk of bias, imprecision, inconsistency, indirectness, and magnitude of effect, will be summarised in summary of findings table (Appendix 5). Based on this information quality of evidence will be rated and detailed information about the

For peer review only - http://bmjopen.bmj.com/site/about/guidelines.xhtml

reason for the quality of evidence rating will be recorded. Outcomes will be categorised as strong or weak according to the quality of the supporting evidence.

Patient and Public Involvement

No patient involved.

ETHICS AND DISSEMINATION

This study is a part of a wider NIHR Global Health Research Unit on Health System Strengthening in Sub-Saharan Africa (ASSET) Sierra Leone project. The results will be communicated and disseminated during meetings with key stakeholders at national, district and community levels, that will be on-going throughout the project. The results will inform the development and implementation of a quality improvement intervention to improve quality of surgical and anaesthesia care in SSACs in general and in Sierra Leone in particular. The results of the review will be published in a peer reviewed journal and presented at national and international conferences. As this is a review of secondary data no formal ethical approval is required.

CONCLUSIONS

To our knowledge this is the first systematic literature review to synthesise the information on HSSIs to improve the quality of hospital-based surgical and anaesthesia care in SSACs. The findings from this systematic review will provide evidence to inform the development, design and implementation of the HSSIs to improve the quality of health care for surgical patients at hospital level in SSACs. It will also provide valuable information for policymakers, healthcare managers and clinicians.

REFERENCES

1. Meara JG, Leather AJM, Hagander L, et al. Global Surgery 2030: Evidence and solutions for achieving health, welfare, and economic development. Lancet. 2015;386(9993):569-624. doi:10.1016/S0140-6736(15)60160-X. (Accessed 19th December 2019).
2. Shrime MG, Bickler SW, Alkire BC, Mock C. Global burden of surgical disease: an estimation from the provider perspective. Lancet Glob Health. 2015;3 Suppl2:S8-9. (Accessed 19th December 2019).

For peer review only - http://bmjopen.bmj.com/site/about/guidelines.xhtml

3. Gosselin RA, Spiegel DA, Coughlin R, Zirkle LG. Injuries: the neglected burden in developing countries. Bull World Health Organ. 2009;87(4):246-a. (Accessed 19th December 2019).

4. Weiser TG, Haynes AB, Molina G, et al. Estimate of the global volume of surgery in 2012: an assessment supporting improved health outcomes. Lancet 2015; 385 (Global Surgery special issue): S11 (Accessed 19th December 2019).

5. Biccard BM, Madiba TE, Kluyts HL, et al. African Surgical Outcomes Study (ASOS) investigators. (Accessed 19th December 2019).

6. Bickler SN, Weiser TG, Kassebaum N, et al. Editors In: Debas HT, Donkor P, Gawande A, Jamison DT, Kruk ME, Mock CN, editors. Source Essential Surgery: Disease Control Priorities, Third Edition (Volume 1). Washington (DC): The International Bank for Reconstruction and Development / The World Bank; 2015 Apr. Chapter 2. (Accessed 19th December 2019).

7. Kruk M E, Gage A D, Arsenault C, et al, High-quality health systems in the Sustainable Development Goals era: time for a revolution The Lancet Global Health, Volume 7, Issue 3, March 2019, Pages e303-e304 (Accessed 19th December 2019).

8. Liberati A, Altman DG, Tetzlaff J, et al. The PRISMA statement for reporting systematic reviews and meta-analyses of studies that evaluate healthcare interventions: explanation and elaboration. *BMJ. 2009 Jul 21; 339():b2700.* (Accessed 19th December 2019).

9. Shamseer L, Moher D, Clarke M, et al. Preferred reporting items for systematic review and meta-analysis protocols (PRISMA-P) 2015: elaboration and explanation. BMJ 2015;349:g7647. (Accessed 19th December 2019).

10. Moher D, Liberati A, Tetzlaff J, et al. Preferred reporting items for systematic reviews and meta-analyses: the PRISMA statement. BMJ 2009;339: b2535. (Accessed 19th December 2019).

11. World Health Organization Everybody's Business: Strengthening Health Systems to Improve Health Outcomes: WHO's Framework for Action 2007 Geneva World health Organization (Accessed 19th December 2019).

12. World Bank. World Bank Country and Lending Groups. 2018. https://datahelpdesk.worldbank.org/knowledgebase/articles/906519-world-bank-country-and-lending-groups (Accessed 19th December 2019).

For peer review only - http://bmjopen.bmj.com/site/about/guidelines.xhtml

13. Bramer W, Rethlefsen M, Kleijnen J, et al. Optimal database combinations for literature searches in systematic reviews: a prospective exploratory study. Systematic reviews 2017; 6:245. (Accessed 19th December 2019).

14. Observational and interventional study design types; an overview. BioMed. 2014; 24(2):199-210 https://www.researchgate.net/publication/263475899_Observational_and_interventional_study_design_types_an_overview. (Accessed 19th December 2019).

15. Ouzzani, M, Hammady H, Fedorowicz Z, et al., Rayyan-a web and mobile app for systematic reviews. Syst Rev, 2016. 5(1): p. 210.

16. Proctor E et al. Outcomes for implementation research: conceptual distinctions, measurement challenges, and research agenda. Adm Policy Ment Health 2011; 38, 2:65-76 (Accessed 19th December 2019).

17. Guyatt 2011a. Guyatt G, Oxman AD, et al. GRADE guidelines: 1. Introduction—GRADE evidence profiles and summary of findings tables. Journal of Clinical Epidemiology 2011; 64: 383–394. (Accessed 19th December 2019).

For peer review only - http://bmjopen.bmj.com/site/about/guidelines.xhtml

Acknowledgments

This study is part of Health System Strengthening in Sub-Saharan Africa (ASSET) and supported by the King's Centrefor Global Health and Health Partnership. The authors thank the library services at King's College London, UK.

Author Statement:

NB, AL and JD conceived the idea for the study and designed the protocol. NB drafted the protocol and is the guarantor of the review. All authors provided feedback on the study design and review methods and contributed to the final manuscript.

Funding:

This systematic review is supported by the NIHR Global Health Research Unit on Health Systems Strengthening in Sub-Saharan Africa, King's College London (GHRU 16/136/54) The funding body had no input into the study design.

Competing interests:
The authors declare no competing interests.

Word count: 2188

Figure 1: Data search and extraction flow diagram

For peer review only - http://bmjopen.bmj.com/site/about/guidelines.xhtml

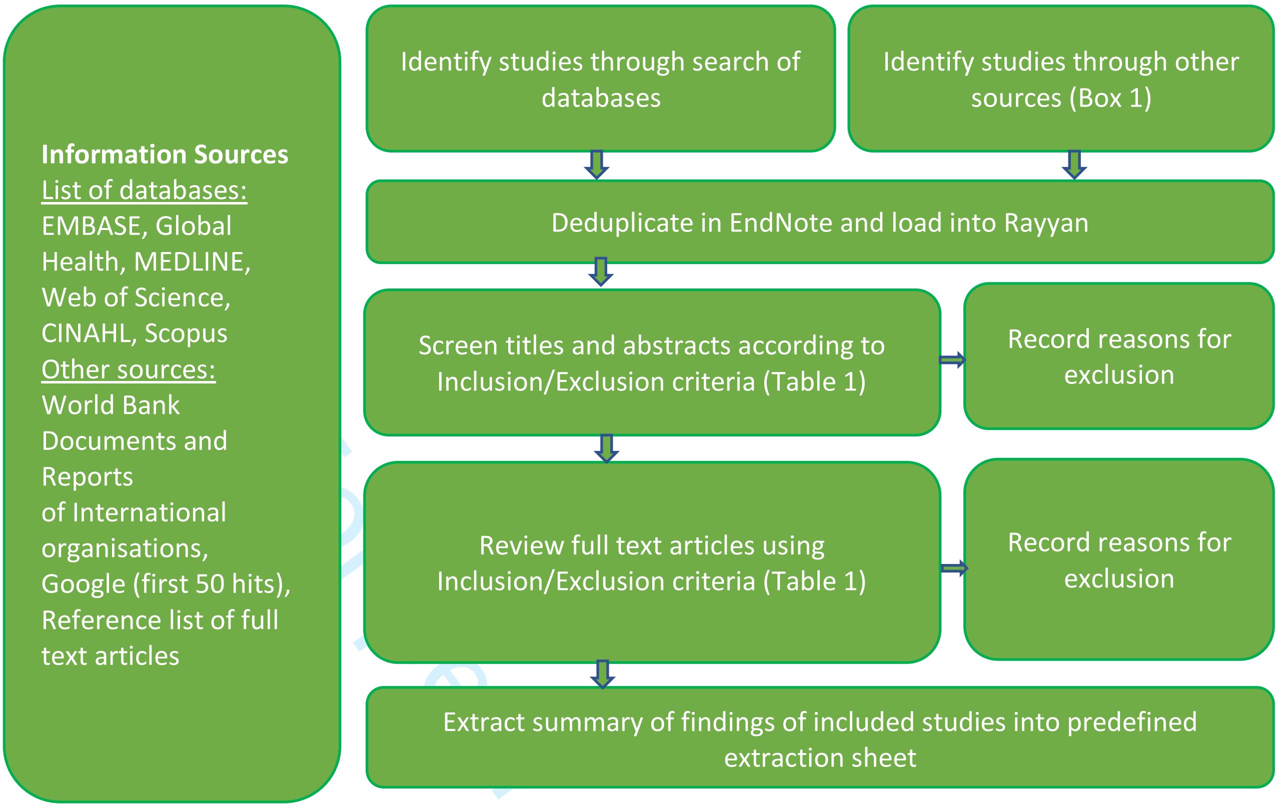

Figure 1: Data search and extraction flow diagram

For peer review only

Supplementary File 1

Improving quality of surgical and anaesthesia care at hospital level in SSACs: a systematic review protocol of health system strengthening interventions.

PRISMA-P (Preferred Reporting Items for Systematic review and Meta-Analysis Protocols) 2015 checklist: recommended items to address in a systematic review protocol*

Section and topic	Item No	Checklist item	Section where each item is addressed in the protocol
**ADMINISTRATIVE INFORMATION**			
Title:			
Identification	1a	Identify the report as a protocol of a systematic review	Title page
Update	1b	If the protocol is for an update of a previous systematic review, identify as such	N/A
Registration	2	If registered, provide the name of the registry (such as PROSPERO) and registration number	Title page
Authors:			
Contact	3a	Provide name, institutional affiliation, e-mail address of all protocol authors; provide physical mailing address of corresponding author	Title page
Contributions	3b	Describe contributions of protocol authors and identify the guarantor of the review	Contributions
Amendments	4	If the protocol represents an amendment of a previously completed or published protocol, identify as such and list changes; otherwise, state plan for documenting important protocol amendments	Methods and Analysis
Support:			
Sources	5a	Indicate sources of financial or other support for the review	Funding
Sponsor	5b	Provide name for the review funder and/or sponsor	Funding
Role of sponsor or funder	5c	Describe roles of funder(s), sponsor(s), and/or institution(s), if any, in developing the protocol	Funding
**INTRODUCTION**			
Rationale	6	Describe the rationale for the review in the context of what is already known	Introduction
Objectives	7	Provide an explicit statement of the question(s) the review will address with reference to participants, interventions, comparators, and outcomes (PICO)	Aim and Objectives
**METHODS**			
Eligibility criteria	8	Specify the study characteristics (such as PICO, study design, setting, time frame) and report characteristics (such	Search strategy, Eligibility

For peer review only

		as years considered, language, publication status) to be used as criteria for eligibility for the review	criteria, Table 1, Appendix 3
Information sources	9	Describe all intended information sources (such as electronic databases, contact with study authors, trial registers or other grey literature sources) with planned dates of coverage	Search strategy, Figure 1, Appendix 2
Search strategy	10	Present draft of search strategy to be used for at least one electronic database, including planned limits, such that it could be repeated	Appendix 1
Study records:			
Data management	11a	Describe the mechanism(s) that will be used to manage records and data throughout the review	Data extraction
Selection process	11b	State the process that will be used for selecting studies (such as two independent reviewers) through each phase of the review (that is, screening, eligibility and inclusion in meta-analysis)	Data extraction
Data collection process	11c	Describe planned method of extracting data from reports (such as piloting forms, done independently, in duplicate), any processes for obtaining and confirming data from investigators	Data extraction
Data items	12	List and define all variables for which data will be sought (such as PICO items, funding sources), any pre-planned data assumptions and simplifications	Appendix 4 and Data Synthesis
Outcomes and prioritization	13	List and define all outcomes for which data will be sought, including prioritization of main and additional outcomes, with rationale	Outcomes
Risk of bias in individual studies	14	Describe anticipated methods for assessing risk of bias of individual studies, including whether this will be done at the outcome or study level, or both; state how this information will be used in data synthesis	Risk of bias
Data synthesis	15a	Describe criteria under which study data will be quantitatively synthesised	Data Synthesis
	15b	If data are appropriate for quantitative synthesis, describe planned summary measures, methods of handling data and methods of combining data from studies, including any planned exploration of consistency (such as I^2, Kendall's τ)	Appendix 5
	15c	Describe any proposed additional analyses (such as sensitivity or subgroup analyses, meta-regression)	Data Synthesis
	15d	If quantitative synthesis is not appropriate, describe the type of summary planned	Data Synthesis
Meta-bias(es)	16	Specify any planned assessment of meta-bias(es) (such as publication bias across studies, selective reporting within studies)	Data Synthesis
Confidence in cumulative evidence	17	Describe how the strength of the body of evidence will be assessed (such as GRADE)	Data Synthesis

*** It is strongly recommended that this checklist be read in conjunction with the PRISMA-P Explanation and Elaboration (cite when available) for important clarification on the items. Amendments to a review protocol should be tracked and dated.** **The copyright for PRISMA-P (including checklist) is held by the PRISMA-P Group and is distributed under a Creative Commons Attribution Licence 4.0.**

From: Shamseer L, Moher D, Clarke M, Ghersi D, Liberati A, Petticrew M, Shekelle P, Stewart L, PRISMA-P Group. Preferred reporting items for systematic review and meta-analysis protocols (PRISMA-P) 2015: elaboration and explanation. BMJ. 2015 Jan 2;349(jan02 1):g7647.

For peer review only

Appendix1: Search strategies for MEDLINE

1) Surgical or anaesthesia ;2) quality improvement hospital level interventions;3) Sub-Saharan African countries (SSACs)

Search String 1	Search String 2	Search String 3
surg* OR surgic* OR an?esthes* OR operat* OR pre?operat* OR post?operat* OR obstetr* OR laparotomy OR ambulatory surgical procedures/ or exp laparotomy/ or exp minor surgical procedures/ or procedures/ or obstetric surgical procedures/ or exp procedures/ or exp perioperative care/ or perioperative exp period/ OR exp General Surgery/ OR exp Anesthetics, General/ or exp Anesthetics, Inhalation/ or exp Anesthetics, Combined/ or exp Anesthetics/ or exp	Hospital* OR in?patient* OR ward* OR (intensive adj1 care*).mp OR exp Hospitals/ OR exp Intensive Care Units, Pediatric/ or exp Hospital Units/ or exp Mobile Health Units/ or exp Coronary Care Units/ or exp Intensive Care Units/ or exp Colony-Forming Units Assay/ or exp Burn Units/ or exp Intensive Care Units, Neonatal/ OR exp inpatient/ OR (Operat* adj2 (room* or theatre)).mp<br>AND (any of the following)<br>intersectoral collaboration/ or efficiency, organizational/ or "facilities and services utilization"/ or hospital information systems/ or hospital-patient relations/ or database management systems/ or ambulatory care information systems/ or medical order entry systems/ or operating room information systems/ or point-of-care systems/ or "personnel staffing and scheduling information systems"/ or medication systems, hospital/ or patient identification systems/ or health smart cards/ or radio frequency identification device/ or employee discipline/ or employee incentive plans/ or employee performance appraisal/ or staff development/ or time management/<br>advance directive adherence/ or exp clinical competence/ or exp guideline adherence/ or exp patient reported outcome measures/ or exp alert fatigue, health personnel/ or exp benchmarking/ or exp clinical audit/ or exp credentialing/ or exp "facility regulation and control"/ or exp guidelines as topic/ or exp Quality Control/ or exp "Quality of Life"/ or exp Quality-Adjusted Life Years/<br>OR "Reminder Systems"/ or "Total Quality Management"/ or exp "Patient Education"/ or "Health Education"/ or exp "Consumer Health Information"/ or "Critical Pathways"/ or "Education, Medical, Continuing"/ or exp "Inservice Training"/ or "Guideline Adherence"/ or "Clinical Competence"/ or "Peer Review"/ or exp "Medical Audit"/ OR OR patient education.mp OR critical pathway*.mp OR clinical pathway*.mp OR care pathway*.mp OR (continuing adj2 education).mp OR (inservice or in service).mp OR (staff adj3 train*).mp OR audit* or feedback* OR exp Medical Record Linkage/ OR exp Patient Safety/ or exp Safety Management/ OR "task performance and analysis"/ or "time and motion studies"/ or work simplification/ OR	sub?Saharan Africa*.mp OR Angola or Benin or Bolivia or "Burkina Faso" or Burundi or "Cabo Verde" or Cameroon or "Central African Republic" or Chad or Comoros or Congo or "Democratic Republic of the Congo" or DRC or "Republic of the Congo" or "Cote d'Ivoire" or "Ivory Coast" or "Equatorial Guinea" or Eritrea or Eswatini or Ethiopia or Gabon or Gambia or Ghana or Guinea or "Guinea?Bissau" or Kenya or Lesotho or Liberia or Madagascar or Malawi or Mali or Mauritania or Mauritius or Mozambique or Namibia or Niger or

For peer review only

Anesthetics, Local/ or exp Anesthetics, Intravenous/ or exp Anesthetics, Dissociative/ OR exp Obstetrics/	"patient risk management".mp OR "clinical documentation" OR "nursing documentation" OR (clinical adj2 record*) OR (patient* adj2 record*) OR (hospital adj2 documentation) OR (nursing process) OR (Patient adj2 care plan*) OR (nurs* adj2 documentation adj2 framework*) OR OR (audit adj3 feedback).mp OR (safety checklist* or safety check list*).mp, (clinical adj2 record*).mp, (patient* adj2 record*).mp, (clinical adj2 documentation).mp, (data adj2 qualit*).mp, (hospital adj2 documentation).m AND exp Quality Improvement/ OR "Outcome and Process Assessment"/ or "Outcome Assessment"/ or "Process Assessment"/ or "Quality of Health Care"/ or "Quality Assurance"/ or "Quality Improvement"/ or "Quality Indicators"/ or "Management Quality Circles" OR (outcome* adj3 assessment*).mp OR (process* adj3 assessment*).mp OR quality assurance.mp OR quality improvement.mp OR (improvement adj intervention*).mp OR (improvement adj program*).mp OR (improvement adj initiative*).mp OR (process* adj improvement).mp OR quality indicator*.mp OR quality circle*.mp OR (total quality management or six sigma*).mp OR (program* adj3 effectiveness).mp OR (program* adj3 evaluation*).mp OR "Program Evaluation"/ or quality* or safet*	Nigeria or Rwanda or "Sao Tome and Principe" or Senegal or "Sierra Leone" or Seychelles or Somalia or "South Africa" or "South Sudan" or Sudan or Tanzania or Togo or Uganda or Zambia or Zimbabwe

*Include USA spelling, plurals, and synonym

For peer review only

Appendix2: Database literature search log.

Date	Database Name	Search Terms	Search Limits	Results	Comments

Apendix3: Systematic Review Abstract and Full Text Screening Criteria

Topic	Criteria	Yes	Unclear	No
Type of article	Is this a Peer-reviewed research articles			
Health Intervention care setting (HICS)	Is this intervention in hospital-based setting			
Type of condition	1.Does this study relate to any surgical and anaesthesia care presentation			
	2.Does this study relate to trauma/injury care, cosmetic surgery or sport medicine?			
Outcome	Does this study report Outcomes-Clinicla, Process, Implementation			
Subject of study	Does this study about Medical production, vaccines and technologies			

Instruction

- Include Abstract and Find Full Text if Type of article (Yes) AND HICS (Yes) AND Type of conditions 1 (Yes) AND Type of conditions 2 (No/Unclear) AND Outcome (Yes) and Subject Study (No)
- Exclude Abstract and Full text if Type of article (Unclear/No) OR HICS (Unclear/No) OR Type of conditions 1 (Unclear/No) OR Type of conditions 2 (Yes) OR Outcome (No) OR Subject Study (Yes)

For peer review only

Appendix 4: Systematic Review Data Extraction Form

Variable	Response
**Reference**	[text]
**Year**	[number]
**Language**	[text]
**Location(s)**	[text]
**Type of publication**	[Grey (1) /Postgrad Dissertation (2)/Published- peer reviewed (3)/Non-research report (4)]
**Journal**	[text]
**Journal Field**	[text]
**Name of low and middle income country**	[text]
**Brief description of study**	[text]
**Surgical and anaesthesia care Intervention**	[yes/no]
**Integrated into routine health services**	[yes/no]
**Health care setting**	[Tertiary (1)/ Secondary Hospital (2)/ Level-hospital department (triage, surgical theatre) (3)]
**Study Methodology**	[Qualitative (1)/quantitative (2)]/ mixed (3)
**Study design**	[RCT, Observational, cohort, quazi-experimental, etc)]
**Describes Evaluation of the intervention**	[retrospective/prospective/neither]
**Type of health intervention**	[Service delivery (1)/Health Workforce (2)/Information (3)/ Financing (4)/Leadership/Governance (5)]
**Population size/Sample size**	
**Primary clinical outcome**	[mortality Yes/No]
**Result of Primary clinical Outcome**	[test]
**Secondary Clinical outcome collected and assessed**	[major or minor complications, SSI, etc]
**Process outcomes collected and assessed**	[text]
**Implementation outcome collected and assessed**	[one of 8 as defined by Proctor]
**Other outcomes collected and assessed**	[text]
**Describes design of intervention**	[text] e.g System thinking, TOC, LF
**Describes Implementation of intervention**	[text] e.g Fidelity, Adoption
**Type of impact**	[text]
**Detail on statistical analysis**	[text]
**Potential Biases or concerns**	[text]
**Cost/source of funding for intervention**	
**Other points or key lessons for reflection from the authors**	[text]
**Notes**	[text]

For peer review only

For peer review only

Appendix 5: GRADE Quality assessment criteria *Journal of Clinical Epidemiology* 2011 64, 383-394DOI: (10.1016/j.jclinepi.2010.04.026) Copyright © 2011 Elsevier Inc. Terms and Conditions

Study Design	Quality of Evidence	Lower if	Higher if
Randomized trial →	High	Risk of bias<br>-1 Serious<br>-2 Very serious<br><br>Inconsistency<br>-1 Serious<br>-2 Very serious<br><br>Indirectness<br>-1 Serious<br>-2 Very serious<br><br>Imprecision<br>-1 Serious<br>-2 Very serious<br><br>Publication bias<br>-1 Likely<br>-2 Very likely	Large effect<br>+1 Large<br>+2 Very large<br><br>Dose response<br>+1 Evidence of a gradient<br><br>All plausible confounding<br>+1 Would reduce a demonstrated effect or<br><br>+1 Would suggest a spurious effect when results show no effect
	Moderate		
Observational study →	Low		
	Very low		

review only

Improving quality of surgical and anaesthesia care at hospital level in Sub-Saharan Africa: a systematic review protocol of health system strengthening interventions.

Journal:	*BMJ Open*
Manuscript ID	bmjopen-2019-036615.R2
Article Type:	Protocol
Date Submitted by the Author:	04-Mar-2020
Complete List of Authors:	Brima, Nataliya; King's College London, King's Centre for Global Health & Health Partnerships<br>Davies, Justine ; Kings College London , Global Health<br>Leather, Andy; King's Centre for Global Health and Health Partnerships
**Primary Subject Heading**:	Surgery
Secondary Subject Heading:	Surgery
Keywords:	Quality in health care < HEALTH SERVICES ADMINISTRATION & MANAGEMENT, PUBLIC HEALTH, SURGERY

SCHOLARONE™
Manuscripts

I, the Submitting Author has the right to grant and does grant on behalf of all authors of the Work (as defined in the below author licence), an exclusive licence and/or a non-exclusive licence for contributions from authors who are: i) UK Crown employees; ii) where BMJ has agreed a CC-BY licence shall apply, and/or iii) in accordance with the terms applicable for US Federal Government officers or employees acting as part of their official duties; on a worldwide, perpetual, irrevocable, royalty-free basis to BMJ Publishing Group Ltd ("BMJ") its licensees and where the relevant Journal is co-owned by BMJ to the co-owners of the Journal, to publish the Work in this journal and any other BMJ products and to exploit all rights, as set out in our licence.

The Submitting Author accepts and understands that any supply made under these terms is made by BMJ to the Submitting Author unless you are acting as an employee on behalf of your employer or a postgraduate student of an affiliated institution which is paying any applicable article publishing charge ("APC") for Open Access articles. Where the Submitting Author wishes to make the Work available on an Open Access basis (and intends to pay the relevant APC), the terms of reuse of such Open Access shall be governed by a Creative Commons licence – details of these licences and which Creative Commons licence will apply to this Work are set out in our licence referred to above.

Other than as permitted in any relevant BMJ Author's Self Archiving Policies, I confirm this Work has not been accepted for publication elsewhere, is not being considered for publication elsewhere and does not duplicate material already published. I confirm all authors consent to publication of this Work and authorise the granting of this licence.

For peer review only

Systematic Review protocol

Improving quality of surgical and anaesthesia care at hospital level in Sub-Saharan Africa: a systematic review protocol of health system strengthening interventions.

PROSPERO Registration number: CRD42019125570

Nataliya Brima[1], Justine Davies[2], Andy Leather[1]

Corresponding author:

Mrs Nataliya Brima
[1]King's Centre for Global Health and Health Partnerships
School of Population Health and Environmental Sciences
Faculty of Life Sciences and Medicine
King's College London
Suite 2.13, Weston Education Centre
Cutcombe Road
London
SE5 9RJ
UK
nataliya.brima@kcl.ac.uk
+447930271138

Prof Justine Davies
[2]Institute for Applied Health Research, University of Birmingham, UK
J.Davies.6@bham.ac.uk

Mr Andy Leather
[1]King's Centre for Global Health and Health Partnerships, School of Population Health and Environmental Sciences, Faculty of Life Sciences and Medicine, King's College London, UK
andy.leather@kcl.ac.uk

Key words: surgical and anaesthesia care, improving quality of care, health system strengthening, Sub-Saharan Africa

For peer review only

For peer review only - http://bmjopen.bmj.com/site/about/guidelines.xhtml

Abstract

Introduction: Over 5 billion people in the world do not have access to safe, affordable surgical and anaesthesia care when needed. In order to improve health outcomes in patients with surgical conditions, both access to care and the quality of care needs to be improved. A recent commission on high quality health highlighted that poor-quality care is now a bigger barrier than non-utilisation of the health system for reducing mortality.

Aim: To carry out a systematic review to provide an evidence-based summary of hospital-based interventions associated with improved quality of surgical and anaesthesia care in Sub-Saharan African countries (SSACs).

Methods and Analysis: Three search strings: 1) surgery and anaesthesia, 2) quality improvement hospital-based interventions, 3) SSACs will be combined.

The following databases: EMBASE, Global Health, MEDLINE, CINAHL, Web of Science and Scopus will be searched. Further relevant studies will be identified from national and international health organisations and publications and reference lists of all selected full text articles.

The review will include all type of original articles in English published between 2008 and 2019. Article screening, data extraction and assessment of methodological quality will be done by two reviewers independently and any disputes will be resolved by a third reviewer or team consensus. Three types of outcomes will be collected including clinical, process and implementation outcomes. The primary outcome will be mortality. Secondary outcomes will include other clinical outcomes (major and minor complications), as well as process and implementation outcomes. Descriptive statistics and outcomes will be summarised and discussed. For the primary outcome the methodological rigour will be assessed.

Ethics and Dissemination: The results will be published in a peer reviewed open access journal and presented at national and international conferences. As this is a review of secondary data no formal ethical approval is required.

Protocol PROSPERO registration number CRD42019125570

Strengths and Limitations of this study

- A comprehensive search strategy for original articles and grey literature both published and unpublished was created and included.
- The inclusion of all fields of surgery in the search strategy will ensure that no health system strengthening interventions (HSSI) implemented within Sub-Saharan Africa (SSA) region are missed.
- It is possible that the included studies (and reported data) will be too few to address the objectives of this study.

For peer review only

- There is the potential that the data retrieved from different studies may not be applicable to every SSA country context due to differences in the healthcare system and therefore will need to be carefully adapted to the context of another country.
- Although every effort will be made to address the publication bias, we acknowledge that unsuccessful quality improvement efforts and unwanted side-effects are rarely published, and as a result this review may not reflect the real current experience of failed attempts in the field of quality improvement of surgical care.

INTRODUCTION

The urgent need for surgical and anaesthesia care continues to be neglected, with over five billion people worldwide being deprived access to safe, affordable and timely surgical and anaesthesia care when needed [1]. SSACs are disproportionally affected where 93% of the population do not have access to basic surgical care [1]. Mortality and morbidity from treatable surgical diseases is high and continues to grow. In 2010, an estimated 32.9% of all lives lost were due to surgical burden of disease [2]. Untreated surgical diseases are among the top 15 causes of physical disability worldwide, with injuries accounting for 15% of all ill-health worldwide and 90% of deaths from injuries occurring in low- or middle- income countries (LMICs) [3], 85% of all SSACs are classified as either low- or middle- income countries. The poorest countries account for 34·8% of the global population, yet annually, only 6% of surgical procedures worldwide take place in these countries [4] and SSA region has the greatest unmet need for surgical procedures [1]. Surgical patients in Africa are twice as likely to die after surgeries, despite being younger, lower-risk profile and developing fewer complications, compared to the global average [5].

Annually, 1.8 million deaths in LMICs could be surgically averted, with 1.4M treatable by basic surgical care delivered at first-level hospitals and 0.4M treatable by advanced surgical care delivered in specialised facilities [6]. This will require strong health systems with effective referral from community to primary care and onwards to secondary and tertiary hospital care [1] in order to address the barriers in accessing quality care. Recently, the Lancet Global Health Commission on High Quality Health Systems in SDG Era highlighted the low quality and performance of many LMIC health systems and placed emphasis on the prioritisation of high-quality care to reduce avoidable mortality and morbidity [7].

In this systematic review we will identify, critically appraise and synthesise the existing published evidence on health system interventions that have focused on quality surgical and anaesthesia care in hospitals in SSACs. This study will inform future efforts to improve the quality of hospital-based care for patients requiring surgical and anaesthesia care.

For peer review only - http://bmjopen.bmj.com/site/about/guidelines.xhtml

METHODS AND ANALYSIS

The systematic review will be conducted, and the data reported in accordance with the Preferred Reporting Items for Systematic Review and Meta-Analysis (PRISMA) statement [8]. This protocol was developed following the PRISMA Protocol (PRISMA-P) 2015 checklist [9, 10], (Supplementary File 1). Amendments to the protocol are not anticipated but will be reported in the publication of the results, should they occur.

Aim

To provide an evidence-based summary of hospital-based interventions associated with improved quality of surgical and anaesthesia care in SSACs.

Objectives:

- To systematically identify studies that evaluate interventions aimed at improving quality of surgical and anaesthesia care at hospital level in SSACs.
- To capture, categorise and assess the study characteristics (setting and intervention).
- To capture and categorise all outcomes reported under the following categories – clinical, process, implementation or other
- To assess methodological rigour of studies with mortality as a primary outcome (which is defined as mortality within 30 days defined for this review).
- To record whether process and implementation outcomes were assessed

Search strategy

The search string strategy was developed by the main author and checked by a research librarian for errors and omissions. During the development process the search was tested and retested to ensure optimal sensitivity and specificity and search terms refined accordingly. The search strategy was developed in MEDLINE (Appendix 1). The search terms used intended to cover a wide range of studies that evaluated interventions aimed at improving the quality of surgical and anaesthesia care at hospital level in SSACs. The three distinct search strings that were combined to identify these studies are: (1) surgical and anaesthesia care, (2) quality improvement hospital-based interventions, (3) SSACs.

Surgical and anaesthesia care will include all branches of surgery (cardiothoracic, ENT, general, neonatal, obstetric, ophthalmic, orthopaedic, paediatric and urology), including

For peer review only - http://bmjopen.bmj.com/site/about/guidelines.xhtml

operative and non-operative aspects of care, on presumption that any branch of surgery may have relevant transferable interventions within a low resource setting. For the purpose of this review only studies that explicitly refer to a surgical procedure or a surgical patient will be included. Trauma (both explicitly focusing or included as part of the intervention) will be excluded, as it will be covered in a separate systematic review.

For the purposes of this review HSSI is defined as any actions or strategies taken to improve the quality of health care delivery and patient outcomes. This will include studies that either strengthen the whole surgical system within a hospital or contribute to one aspect of surgical and anaesthesia care. The WHO Health System Framework will be used to categorise HSSIs [11].

Hospital-based interventions are defined as any intervention that is delivered within a tertiary/teaching/national or a secondary/district/regional care hospital environment, aimed at improving quality of care, that directly or indirectly involves care delivery to patients or by staff. Both hospital-wide (hospital documentation, early warning scores, intensive care outreach) and individual departmental (emergency department, surgical wards, theatre, HDU, ITU, outpatient, radiology) interventions will be included.

The term SSACs will include all countries listed as Sub-Saharan Africa counties by the World Bank for the 2019 fiscal year [12].

To combine terms within each search string the Boolean operator 'OR' was used. To incorporate all variations of the same term, truncation and wildcards was employed. Medical Subject Headings (MeSH) terms and free text was also utilised. To focus the search on these required terms, phrase and adjacency searching, using proximity operator filters and limits was exploited. Any additional key terms identified during the literature search were added to the appropriate string to ensure it was maximally inclusive. The three strings were then combined using the Boolean operator 'AND'.

This search strategy will be adapted to EMBASE, Global Health, CINAHL, Web of Science databases. A reference list of all included articles will be hand-searched for further relevant studies.

To identify unpublished studies the Scopus literature databases will be searched. In addition, first 50 hits from Google search, documentation and reports of relevant national and international health organisations such as Comic Relief, DFID, Grand Challenges

For peer review only - http://bmjopen.bmj.com/site/about/guidelines.xhtml

Canada, the Bill and Melinda Gates Foundation, HIVOS, World Vision, the Robert Wood Johnson foundation, UNISEF, GOALS will be searched.

The choice of information sources for this search were made based on the recommendation from the current literature, to ensure the optimal combination of databases and other search resources available needed to guaranty adequate and efficient coverage [13].

The search process will be recorded using a Database literature search log (Appendix 2).

Eligibility criteria

All eligible articles published in English will be included, from 2008 to 2019. The inclusion and exclusion criteria are illustrated in Table 1 below.

Table 1: Inclusion and Exclusion criteria

	**Include**	**Exclude**
Type of Article	All Peer-reviewed research articles<br><br>Non-research reports from national or international health organisations, dissertations/theses, books/book chapters, conference abstracts, and research in progress from grey literature	Unstructured reviews or overviews, theoretical papers, commentaries or opinion papers,<br><br>Case studies, audits, editorials/ letters/ comments, newspapers/ trade journals, literature reviews<br><br>Guidelines, strategies and policies from national or international health organisations
Type of conditions	Any surgical and anaesthesia care (operative or non-operative); type of presentation (elective or emergency); sub-speciality surgical or anaesthesia care (including peri-operative medicine and pain management).	Trauma/injury care<br><br>Studies on cosmetic and aesthetic surgical care and sports medicine
Type of population	General population<br><br>Population with specific surgical diseases or conditions<br><br>Adults, neonatal and children's surgery	Animals

For peer review only - http://bmjopen.bmj.com/site/about/guidelines.xhtml

Care setting	Hospital setting, SSACs	Studies that are not conducted in hospital-based settings.<br><br>Studies that took place outside of SSA
Type of design	Interventional studies[1]	Observational studies
Subject of study	Quality improvement of surgical care through the following areas:<br>-Service delivery<br>-Health Workforce<br>-Information<br>-Financing<br>-Leadership/Governance	-Studies that did not assess outcomes.<br>-Medical device production and new clinical technological devices<br>-Introduction of new procedures<br>-Medical products, vaccines and technologies

[1] Interventional studies are often prospective and are specifically tailored to evaluate direct impacts of treatment or preventive measures on disease [14].

Data extraction

All potential articles will be initially collated in Endnote X8 for de-duplication of results. All remaining studies will be loaded into Rayyan online opensource web application [15] and initially screened by title and abstract by a team of two reviewers independently, according to inclusion and exclusion criteria (Table 1) and by using the Systematic Review Abstract and Full Text Screening Criteria form (Appendix 3). All reviewers were appropriately trained in systematic review methods. If either of reviewers includes an abstract, it will be promoted to consideration for full text review. The full text review will be done by the first author (Nataliya Brima) and another reviewer independently, using inclusion and exclusion criteria. Data elements will be extracted into a predefined data extraction form (Appendix 4), which will be piloted before finalising the elements to be included. This includes information on authors, publication dates, country of intervention, health care setting, type of intervention, methodology or design, patient/intervention size, clinical/process/implementation/other outcomes and the impact of the intervention. The data extracted by two reviews will be compared and if any discrepancies arise, consensus will be sought among the study team (Figure 1).

Outcomes

Three type of outcomes- clinical, process and implementation will be collected. The primary outcome for this review will be - mortality (in-hospital perioperative mortality and post

For peer review only - http://bmjopen.bmj.com/site/about/guidelines.xhtml

intervention mortality within 30 days). All other outcomes will be secondary: clinical outcome such as major and minor complications as defined by the Clavien-Dindo Classification (Table 2) and any other clinical outcomes identified during the course of reviewing the manuscripts. Process outcomes will include all outcomes relevant to interventional studies included in this review (waiting times, length of hospital stay, blood availability, etc). Implementation outcomes will be determined using a ‘working taxonomy’ of 8 conceptually distinct implementation outcomes produced by Proctor et al [16] (Table 3). If any other outcomes are reported that cannot fit into the three categories above, they will be recorded under the heading ‘Other outcomes’, described, and categorised, if possible, after discussion amongst authors.

Table 2: Clavien-Dindo Classification of Complications

Grade	Definition
I	Any deviation from the normal post-operative course without the need for pharmacological treatment or surgical, endoscopic and radiological interventions. Allowed therapeutic regimes are as follows: drugs as anti-emetics, anti-pyretics, analgesics, diuretics, electrolytes and physiotherapy. This grade also includes wound infections opened at the bedside.
II	Requiring pharmacological treatment with drugs other than such allowed for grade I complications. Blood transfusions and total parenteral nutrition are also included.
III	Requiring surgical, endoscopic, or radiological intervention.
IIIa	Intervention not under general anaesthesia.
IIIb	Intervention under general anaesthesia.
IV	Life-threatening complication requiring ICU management.
IVa	Single organ dysfunction (including dialysis).
IVb	Multi-organ dysfunction.
V	Death of a patient.

Table 3: Implementation Outcomes

	Implementation Outcome (Proctor et al)	Definition
1	Acceptability	Degree to which the intervention is perceived agreeable, acceptable or satisfactory
2	Adoption	Intention and willingness to apply the intervention
3	Appropriateness	Perceived relevance of the intervention to be relevant to the problem
4	Feasibility	Extent to which the intervention can be successfully applied
5	Fidelity	Extent to which the intervention is completed as originally intended
6	Implementation Cost	Intervention time and cost
7	Penetration	Spread into practice, e.g proportion of eligible patients who received the intervention
8	Sustainability	Extent to which a new intervention routinely is used in practice

For peer review only - http://bmjopen.bmj.com/site/about/guidelines.xhtml

Risk Of Bias

Risk of bias of individual studies and publication bias will be addressed by designing a thorough search strategy to find relevant studies. The search strategy will be assessed by checking the results against known articles which are relevant. To address publication bias the grey literature will be searched, as described above, to include all relevant studies which are not reported in peer-reviewed journals. The following type of publications will be included: dissertations/theses, books/book chapters, conference abstracts, and research in progress. Also, all potential full text articles will be screened by two reviewers. For studies that reported their main outcome as mortality the reviewers will independently assess the risk of bias as part of the Grading of Recommendations Assessment, Development, and Evaluation (GRADE) system [17] of rating quality of evidence and grading strength of recommendations in systematic reviews.

Disagreements between the reviews over the risk of bias level (serious or very serious) will be resolved by discussion with involvement of a third reviewer where necessary. Due to expected high heterogenicity of the data extracted, a meta-analysis will not be feasible.

Data synthesis

For all outcomes all items that will be collected using predefined collection form (Appendix 4) will be summarised and the methodological approach used in the studies will be identified. Descriptive statistics and narrative synthesis of data will be undertaken to appreciate the diversity of research approaches that are used for assessment of quality of surgical and anaesthesia care within hospital. Those studies that were collected under the heading 'other', we will look for common themes and then compare these themes to our three defined categories of outcomes.

In addition, the studies reporting mortality as the main outcome of the interventions will be assessed using GRADE criteria for rating quality of evidence and grading strength of recommendations in systematic reviews. [17]. All evidence, that used for rating the quality of evidence and include study design, risk of bias, imprecision, inconsistency, indirectness, and magnitude of effect, will be summarised in summary of findings table (Appendix 5). Based on this information quality of evidence will be rated and detailed information about the reason for the quality of evidence rating will be recorded. Outcomes will be categorised as strong or weak according to the quality of the supporting evidence.

For peer review only - http://bmjopen.bmj.com/site/about/guidelines.xhtml

For peer review only

Patient and Public Involvement

No patient involved.

ETHICS AND DISSEMINATION

This study is a part of a wider NIHR Global Health Research Unit on Health System Strengthening in Sub-Saharan Africa (ASSET) Sierra Leone project. The results will be communicated and disseminated during meetings with key stakeholders at national, district and community levels, that will be on-going throughout the project. The results will inform the development and implementation of a quality improvement intervention to improve quality of surgical and anaesthesia care in SSACs in general and in Sierra Leone in particular. The results of the review will be published in a peer reviewed journal and presented at national and international conferences. As this is a review of secondary data no formal ethical approval is required.

To our knowledge this is the first systematic literature review to synthesise the information on HSSIs to improve the quality of hospital-based surgical and anaesthesia care in SSACs. The findings from this systematic review will provide evidence to inform the development, design and implementation of the HSSIs to improve the quality of health care for surgical patients at hospital level in SSACs. It will also provide valuable information for policymakers, healthcare managers and clinicians.

REFERENCES

1. Meara JG, Leather AJM, Hagander L, et al. Global Surgery 2030: Evidence and solutions for achieving health, welfare, and economic development. Lancet. 2015;386(9993):569-624. doi:10.1016/S0140-6736(15)60160-X. (Accessed 19th December 2019).
2. Shrime MG, Bickler SW, Alkire BC, Mock C. Global burden of surgical disease: an estimation from the provider perspective. Lancet Glob Health. 2015;3 Suppl2:S8-9. (Accessed 19th December 2019).
3. Gosselin RA, Spiegel DA, Coughlin R, Zirkle LG. Injuries: the neglected burden in developing countries. Bull World Health Organ. 2009;87(4):246-a. (Accessed 19th December 2019).

For peer review only - http://bmjopen.bmj.com/site/about/guidelines.xhtml

4. Weiser TG, Haynes AB, Molina G, et al. Estimate of the global volume of surgery in 2012: an assessment supporting improved health outcomes. Lancet 2015; 385 (Global Surgery special issue): S11 (Accessed 19th December 2019).

5. Biccard BM, Madiba TE, Kluyts HL, et al. African Surgical Outcomes Study (ASOS) investigators. (Accessed 19th December 2019).

6. Bickler SN, Weiser TG, Kassebaum N, et al. Editors In: Debas HT, Donkor P, Gawande A, Jamison DT, Kruk ME, Mock CN, editors. Source Essential Surgery: Disease Control Priorities, Third Edition (Volume 1). Washington (DC): The International Bank for Reconstruction and Development / The World Bank; 2015 Apr. Chapter 2. (Accessed 19th December 2019).

7. Kruk M E, Gage A D, Arsenault C, et al, High-quality health systems in the Sustainable Development Goals era: time for a revolution The Lancet Global Health, Volume 7, Issue 3, March 2019, Pages e303-e304 (Accessed 19th December 2019).

8. *Liberati A, Altman DG, Tetzlaff J, et al.* The PRISMA statement for reporting systematic reviews and meta-analyses of studies that evaluate healthcare interventions: explanation and elaboration. *BMJ. 2009 Jul 21; 339():b2700.* (Accessed 19th December 2019).

9. Shamseer L, Moher D, Clarke M, et al. Preferred reporting items for systematic review and meta-analysis protocols (PRISMA-P) 2015: elaboration and explanation. BMJ 2015;349:g7647. (Accessed 19th December 2019).

10. Moher D, Liberati A, Tetzlaff J, et al. Preferred reporting items for systematic reviews and meta-analyses: the PRISMA statement. BMJ 2009;339: b2535. (Accessed 19th December 2019).

11. World Health Organization Everybody's Business: Strengthening Health Systems to Improve Health Outcomes: WHO's Framework for Action 2007 Geneva World health Organization (Accessed 19th December 2019).

12. World Bank. World Bank Country and Lending Groups. 2018. https://datahelpdesk.worldbank.org/knowledgebase/articles/906519-world-bank-country-and-lending-groups (Accessed 19th December 2019).

13. Bramer W, Rethlefsen M, Kleijnen J, et al. Optimal database combinations for literature searches in systematic reviews: a prospective exploratory study. Systematic reviews 2017; 6:245. (Accessed 19th December 2019).

14. Observational and interventional study design types; an overview. BioMed. 2014; 24(2):199-

For peer review only - http://bmjopen.bmj.com/site/about/guidelines.xhtml

210 https://www.researchgate.net/publication/263475899_Observational_and_interventional_study_design_types_an_overview. (Accessed 19th December 2019).

15. Ouzzani, M, Hammady H, Fedorowicz Z, et al., Rayyan-a web and mobile app for systematic reviews. Syst Rev, 2016. 5(1): p. 210.
16. Proctor E et al. Outcomes for implementation research: conceptual distinctions, measurement challenges, and research agenda. Adm Policy Ment Health 2011; 38, 2:65-76 (Accessed 19th December 2019).
17. Guyatt 2011a. Guyatt G, Oxman AD, et al. GRADE guidelines: 1. Introduction—GRADE evidence profiles and summary of findings tables. Journal of Clinical Epidemiology 2011; 64: 383–394. (Accessed 19th December 2019).

For peer review only

Acknowledgments

This study is part of Health System Strengthening in Sub-Saharan Africa (ASSET) and supported by the King's Centrefor Global Health and Health Partnership. The authors thank the library services at King's College London, UK.

Author Statement:

NB, AL and JD conceived the idea for the study and designed the protocol. NB drafted the protocol and is the guarantor of the review. All authors provided feedback on the study design and review methods and contributed to the final manuscript.

Funding:

This systematic review is supported by the NIHR Global Health Research Unit on Health Systems Strengthening in Sub-Saharan Africa, King's College London (GHRU 16/136/54) The funding body had no input into the study design.

Competing interests:
The authors declare no competing interests.

Word count: 2188

Figure 1: Data search and extraction flow diagram

For peer review only - http://bmjopen.bmj.com/site/about/guidelines.xhtml

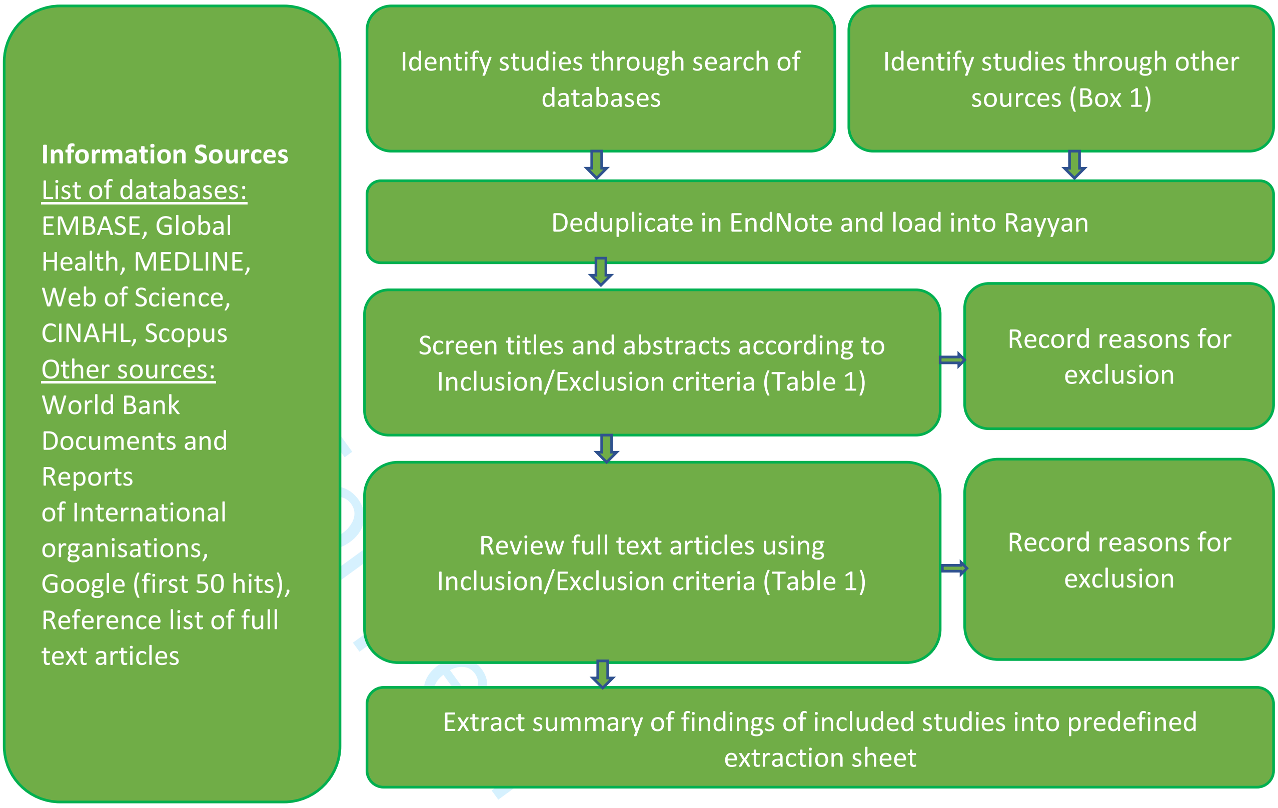

Figure 1: Data search and extraction flow diagram

Supplementary File 1

Improving quality of surgical and anaesthesia care at hospital level in SSACs: a systematic review protocol of health system strengthening interventions.

PRISMA-P (Preferred Reporting Items for Systematic review and Meta-Analysis Protocols) 2015 checklist: recommended items to address in a systematic review protocol*

Section and topic	Item No	Checklist item	Section where each item is addressed in the protocol
**ADMINISTRATIVE INFORMATION**			
Title:			
Identification	1a	Identify the report as a protocol of a systematic review	Title page
Update	1b	If the protocol is for an update of a previous systematic review, identify as such	N/A
Registration	2	If registered, provide the name of the registry (such as PROSPERO) and registration number	Title page
Authors:			
Contact	3a	Provide name, institutional affiliation, e-mail address of all protocol authors; provide physical mailing address of corresponding author	Title page
Contributions	3b	Describe contributions of protocol authors and identify the guarantor of the review	Contributions
Amendments	4	If the protocol represents an amendment of a previously completed or published protocol, identify as such and list changes; otherwise, state plan for documenting important protocol amendments	Methods and Analysis
Support:			
Sources	5a	Indicate sources of financial or other support for the review	Funding
Sponsor	5b	Provide name for the review funder and/or sponsor	Funding
Role of sponsor or funder	5c	Describe roles of funder(s), sponsor(s), and/or institution(s), if any, in developing the protocol	Funding
**INTRODUCTION**			
Rationale	6	Describe the rationale for the review in the context of what is already known	Introduction
Objectives	7	Provide an explicit statement of the question(s) the review will address with reference to participants, interventions, comparators, and outcomes (PICO)	Aim and Objectives
**METHODS**			
Eligibility criteria	8	Specify the study characteristics (such as PICO, study design, setting, time frame) and report characteristics (such	Search strategy, Eligibility

For peer review only

		as years considered, language, publication status) to be used as criteria for eligibility for the review	criteria, Table 1, Appendix 3
Information sources	9	Describe all intended information sources (such as electronic databases, contact with study authors, trial registers or other grey literature sources) with planned dates of coverage	Search strategy, Figure 1, Appendix 2
Search strategy	10	Present draft of search strategy to be used for at least one electronic database, including planned limits, such that it could be repeated	Appendix 1
Study records:			
Data management	11a	Describe the mechanism(s) that will be used to manage records and data throughout the review	Data extraction
Selection process	11b	State the process that will be used for selecting studies (such as two independent reviewers) through each phase of the review (that is, screening, eligibility and inclusion in meta-analysis)	Data extraction
Data collection process	11c	Describe planned method of extracting data from reports (such as piloting forms, done independently, in duplicate), any processes for obtaining and confirming data from investigators	Data extraction
Data items	12	List and define all variables for which data will be sought (such as PICO items, funding sources), any pre-planned data assumptions and simplifications	Appendix 4 and Data Synthesis
Outcomes and prioritization	13	List and define all outcomes for which data will be sought, including prioritization of main and additional outcomes, with rationale	Outcomes
Risk of bias in individual studies	14	Describe anticipated methods for assessing risk of bias of individual studies, including whether this will be done at the outcome or study level, or both; state how this information will be used in data synthesis	Risk of bias
Data synthesis	15a	Describe criteria under which study data will be quantitatively synthesised	Data Synthesis
	15b	If data are appropriate for quantitative synthesis, describe planned summary measures, methods of handling data and methods of combining data from studies, including any planned exploration of consistency (such as I^2, Kendall's τ)	Appendix 5
	15c	Describe any proposed additional analyses (such as sensitivity or subgroup analyses, meta-regression)	Data Synthesis
	15d	If quantitative synthesis is not appropriate, describe the type of summary planned	Data Synthesis
Meta-bias(es)	16	Specify any planned assessment of meta-bias(es) (such as publication bias across studies, selective reporting within studies)	Data Synthesis
Confidence in cumulative evidence	17	Describe how the strength of the body of evidence will be assessed (such as GRADE)	Data Synthesis

*** It is strongly recommended that this checklist be read in conjunction with the PRISMA-P Explanation and Elaboration (cite when available) for important clarification on the items. Amendments to a review protocol should be tracked and dated.** **The copyright for PRISMA-P (including checklist) is held by the PRISMA-P Group and is distributed under a Creative Commons Attribution Licence 4.0.**

From: Shamseer L, Moher D, Clarke M, Ghersi D, Liberati A, Petticrew M, Shekelle P, Stewart L, PRISMA-P Group. Preferred reporting items for systematic review and meta-analysis protocols (PRISMA-P) 2015: elaboration and explanation. BMJ. 2015 Jan 2;349(jan02 1):g7647.

For peer review only

For peer review only - http://bmjopen.bmj.com/site/about/guidelines.xhtml

Appendix1: Search strategies for MEDLINE

1) Surgical or anaesthesia ;2) quality improvement hospital level interventions;3) Sub-Saharan African countries (SSACs)

Search String 1	Search String 2	Search String 3
surg* OR surgic* OR an?esthes* OR operat* OR pre?operat* OR post?operat* OR obstetr*<br>OR laparotomy OR ambulatory surgical procedures/ or exp laparotomy/ or exp minor surgical procedures/ or procedures/ or obstetric surgical procedures/ or exp procedures/ or exp perioperative care/ or perioperative exp period/ OR<br>exp General Surgery/ OR exp Anesthetics, General/ or exp Anesthetics, Inhalation/ or exp Anesthetics, Combined/ or exp Anesthetics/ or exp	Hospital* OR in?patient* OR ward* OR (intensive adj1 care*).mp OR exp Hospitals/ OR exp Intensive Care Units, Pediatric/ or exp Hospital Units/ or exp Mobile Health Units/ or exp Coronary Care Units/ or exp Intensive Care Units/ or exp Colony-Forming Units Assay/ or exp Burn Units/ or exp Intensive Care Units, Neonatal/ OR exp inpatient/ OR (Operat* adj2 (room* or theatre)).mp<br>AND (any of the following)<br>intersectoral collaboration/ or efficiency, organizational/ or "facilities and services utilization"/ or hospital information systems/ or hospital-patient relations/ or database management systems/ or ambulatory care information systems/ or medical order entry systems/ or operating room information systems/ or point-of-care systems/ or "personnel staffing and scheduling information systems"/ or medication systems, hospital/ or patient identification systems/ or health smart cards/ or radio frequency identification device/ or employee discipline/ or employee incentive plans/ or employee performance appraisal/ or staff development/ or time management/<br>advance directive adherence/ or exp clinical competence/ or exp guideline adherence/ or exp patient reported outcome measures/ or exp alert fatigue, health personnel/ or exp benchmarking/ or exp clinical audit/ or exp credentialing/ or exp "facility regulation and control"/ or exp guidelines as topic/ or exp Quality Control/ or exp "Quality of Life"/ or exp Quality-Adjusted Life Years/<br>OR "Reminder Systems"/ or "Total Quality Management"/ or exp "Patient Education"/ or "Health Education"/ or exp "Consumer Health Information"/ or "Critical Pathways"/ or "Education, Medical, Continuing"/ or exp "Inservice Training"/ or "Guideline Adherence"/ or "Clinical Competence"/ or "Peer Review"/ or exp "Medical Audit"/ OR OR patient education.mp OR critical pathway*.mp OR clinical pathway*.mp OR care pathway*.mp OR (continuing adj2 education).mp OR (inservice or in service).mp OR (staff adj3 train*).mp OR audit* or feedback* OR exp Medical Record Linkage/ OR exp Patient Safety/ or exp Safety Management/ OR "task performance and analysis"/ or "time and motion studies"/ or work simplification/ OR	sub?Saharan Africa*.mp OR Angola or Benin or Bolivia or "Burkina Faso" or Burundi or "Cabo Verde" or Cameroon or "Central African Republic" or Chad or Comoros or Congo or "Democratic Republic of the Congo" or DRC or "Republic of the Congo" or "Cote d'Ivoire" or "Ivory Coast" or "Equatorial Guinea" or Eritrea or Eswatini or Ethiopia or Gabon or Gambia or Ghana or Guinea or "Guinea?Bissau" or Kenya or Lesotho or Liberia or Madagascar or Malawi or Mali or Mauritania or Mauritius or Mozambique or Namibia or Niger or

For peer review only

Anesthetics, Local/ or exp Anesthetics, Intravenous/ or exp Anesthetics, Dissociative/ OR exp Obstetrics/	"patient risk management".mp OR "clinical documentation" OR "nursing documentation" OR (clinical adj2 record*) OR (patient* adj2 record*) OR (hospital adj2 documentation) OR (nursing process) OR (Patient adj2 care plan*) OR (nurs* adj2 documentation adj2 framework*) OR OR (audit adj3 feedback).mp OR (safety checklist* or safety check list*).mp, (clinical adj2 record*).mp, (patient* adj2 record*).mp, (clinical adj2 documentation).mp, (data adj2 qualit*).mp, (hospital adj2 documentation).m AND exp Quality Improvement/ OR "Outcome and Process Assessment"/ or "Outcome Assessment"/ or "Process Assessment"/ or "Quality of Health Care"/ or "Quality Assurance"/ or "Quality Improvement"/ or "Quality Indicators"/ or "Management Quality Circles" OR (outcome* adj3 assessment*).mp OR (process* adj3 assessment*).mp OR quality assurance.mp OR quality improvement.mp OR (improvement adj intervention*).mp OR (improvement adj program*).mp OR (improvement adj initiative*).mp OR (process* adj improvement).mp OR quality indicator*.mp OR quality circle*.mp OR (total quality management or six sigma*).mp OR (program* adj3 effectiveness).mp OR (program* adj3 evaluation*).mp OR "Program Evaluation"/ or quality* or safet*	Nigeria or Rwanda or "Sao Tome and Principe" or Senegal or "Sierra Leone" or Seychelles or Somalia or "South Africa" or "South Sudan" or Sudan or Tanzania or Togo or Uganda or Zambia or Zimbabwe

*Include USA spelling, plurals, and synonym

For peer review only

Appendix2: Database literature search log.

Date	Database Name	Search Terms	Search Limits	Results	Comments

Apendix3: Systematic Review Abstract and Full Text Screening Criteria

Topic	Criteria	Yes	Unclear	No
Type of article	Is this a Peer-reviewed research articles			
Health Intervention care setting (HICS)	Is this intervention in hospital-based setting			
Type of condition	1.Does this study relate to any surgical and anaesthesia care presentation			
	2.Does this study relate to trauma/injury care, cosmetic surgery or sport medicine?			
Outcome	Does this study report Outcomes-Clinicla, Process, Implementation			
Subject of study	Does this study about Medical production, vaccines and technologies			

Instruction

- Include Abstract and Find Full Text if Type of article (Yes) AND HICS (Yes) AND Type of conditions 1 (Yes) AND Type of conditions 2 (No/Unclear) AND Outcome (Yes) and Subject Study (No)
- Exclude Abstract and Full text if Type of article (Unclear/No) OR HICS (Unclear/No) OR Type of conditions 1 (Unclear/No) OR Type of conditions 2 (Yes) OR Outcome (No) OR Subject Study (Yes)

For peer review only

Appendix 4: Systematic Review Data Extraction Form

Variable	Response
**Reference**	[text]
**Year**	[number]
**Language**	[text]
**Location(s)**	[text]
**Type of publication**	[Grey (1) /Postgrad Dissertation (2)/Published- peer reviewed (3)/Non-research report (4)]
**Journal**	[text]
**Journal Field**	[text]
**Name of low and middle income country**	[text]
**Brief description of study**	[text]
**Surgical and anaesthesia care Intervention**	[yes/no]
**Integrated into routine health services**	[yes/no]
**Health care setting**	[Tertiary (1)/ Secondary Hospital (2)/ Level-hospital department (triage, surgical theatre) (3)]
**Study Methodology**	[Qualitative (1)/quantitative (2)]/ mixed (3)
**Study design**	[RCT, Observational, cohort, quazi-experimental, etc)]
**Describes Evaluation of the intervention**	[retrospective/prospective/neither]
**Type of health intervention**	[Service delivery (1)/Health Workforce (2)/Information (3)/ Financing (4)/Leadership/Governance (5)]
**Population size/Sample size**	
**Primary clinical outcome**	[mortality Yes/No]
**Result of Primary clinical Outcome**	[test]
**Secondary Clinical outcome collected and assessed**	[major or minor complications, SSI, etc]
**Process outcomes collected and assessed**	[text]
**Implementation outcome collected and assessed**	[one of 8 as defined by Proctor]
**Other outcomes collected and assessed**	[text]
**Describes design of intervention**	[text] e.g System thinking, TOC, LF
**Describes Implementation of intervention**	[text] e.g Fidelity, Adoption
**Type of impact**	[text]
**Detail on statistical analysis**	[text]
**Potential Biases or concerns**	[text]
**Cost/source of funding for intervention**	
**Other points or key lessons for reflection from the authors**	[text]
**Notes**	[text]

For peer review only

For peer review only

Appendix 5: GRADE Quality assessment criteria *Journal of Clinical Epidemiology* 2011 64, 383-394DOI: (10.1016/j.jclinepi.2010.04.026) Copyright © 2011 Elsevier Inc. Terms and Conditions

Study Design	Quality of Evidence	Lower if	Higher if
Randomized trial →	High	Risk of bias<br>-1 Serious<br>-2 Very serious	Large effect<br>+1 Large<br>+2 Very large
	Moderate	Inconsistency<br>-1 Serious<br>-2 Very serious	Dose response<br>+1 Evidence of a gradient
Observational study →	Low	Indirectness<br>-1 Serious<br>-2 Very serious	All plausible confounding<br>+1 Would reduce a demonstrated effect or<br><br>+1 Would suggest a spurious effect when results show no effect
	Very low	Imprecision<br>-1 Serious<br>-2 Very serious<br><br>Publication bias<br>-1 Likely<br>-2 Very likely	

review only
